# MHC class II complexes sample intermediate states along the peptide exchange pathway

**DOI:** 10.1038/ncomms13224

**Published:** 2016-11-09

**Authors:** Marek Wieczorek, Jana Sticht, Sebastian Stolzenberg, Sebastian Günther, Christoph Wehmeyer, Zeina El Habre, Miguel Álvaro-Benito, Frank Noé, Christian Freund

**Affiliations:** 1 Protein Biochemistry, Institute for Chemistry and Biochemistry, Freie Universität Berlin, Thielallee 63, 14195 Berlin, Germany; 2 Computational Molecular Biology group, Institute for Mathematics, Arnimallee 6, 14195 Berlin, Germany; 3 Institute of Human Virology, University of Maryland School of Medicine, 725 West Lombard Street, Baltimore, Maryland 21201, USA

## Abstract

The presentation of peptide-MHCII complexes (pMHCIIs) for surveillance by T cells is a well-known immunological concept in vertebrates, yet the conformational dynamics of antigen exchange remain elusive. By combining NMR-detected H/D exchange with Markov modelling analysis of an aggregate of 275 microseconds molecular dynamics simulations, we reveal that a stable pMHCII spontaneously samples intermediate conformations relevant for peptide exchange. More specifically, we observe two major peptide exchange pathways: the kinetic stability of a pMHCII's ground state defines its propensity for intrinsic peptide exchange, while the population of a rare, intermediate conformation correlates with the propensity of the HLA-DM-catalysed pathway. Helix-destabilizing mutants designed based on our model shift the exchange behaviour towards the HLA-DM-catalysed pathway and further allow us to conceptualize how allelic variation can shape an individual's MHC restricted immune response.

## Abstract (web summary)

MHCII proteins bind and present both foreign and self-antigens to potentially activate CD4+ T cells via cognate T cell receptors (TCRs) during the adaptive immune response. Here, the authors combine NMR-detected H/D exchange with Markov modelling analysis to shed light on the dynamics of MHCII peptide exchange.

## Graphical abstract

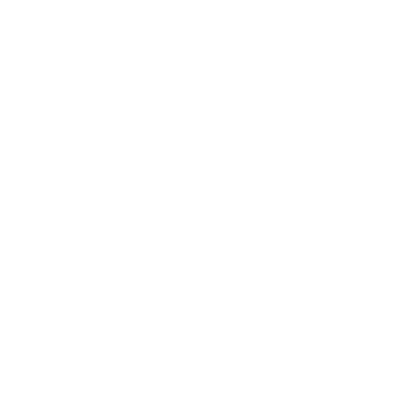

Antigen-specific recognition of peptide-major histocompatibility class II (MHCII) complexes (pMHCIIs) by CD4^+^ T cells represents a key feature of acquired immunity. Professional antigen-presenting cells express a subset of different alleles of heterodimeric MHCII proteins on their surface. These MHCII proteins are capable of binding and presenting both foreign and self-antigens to potentially activate CD4^+^ T cells via cognate T cell receptors as part of the adaptive immune response. The structural hallmarks of antigen binding by MHCII molecules are well understood at the phenomenological level: Optimal occupation of the four main pockets (P_1_, P_4_, P_6_, P_9_) in the MHCII-binding groove by peptide side-chains and concomitant formation of a conserved set of hydrogen bonds mainly between peptide backbone and MHCII side-chains are necessary and sufficient to guarantee high-affinity binding of certain epitopes and their subsequent surveillance by T cells^1^,^2^,^3^. Initially, the class II-associated invariant chain peptide (CLIP) occupies the MHCII-binding groove, but can later be replaced in the endosomal compartments for higher affinity antigens. Here under acidic conditions, the exchange reaction can be catalysed by the non-classical MHCII molecule HLA-DM (DM)^4^,^5^,^6^,^7^,^8^. The propensity to exchange peptides depends on the kinetic stability of an individual pMHCII, as measured by its intrinsic dissociation rate. Stable complexes, however, are also prone to DM-mediated exchange, and it has been postulated that MHCII dynamically samples alternative conformations^9^,^10^,^11^,^12^, which is a major prerequisite for DM susceptibility^13^.

A recently solved pMHCII/DM crystal structure highlights the extensive conformational changes in the P_1_ pocket region required for DM binding of a pMHCII (ref. 14). As yet, details of the molecular mechanism leading to DM binding and peptide exchange remain elusive, but its understanding is of utmost importance for the concepts of immunodominance and autoimmunity^15^,^16^,^17^,^18^,^19^.

Here we show by an integration of structural, biophysical and theoretical methods, that pMHCIIs spontaneously sample transient conformations that have secondary structural features of the DM-stabilized peptide exchange complex. By comparing the stable wild-type pMHCII to a mutant not relying on DM for fast exchange, we reveal the existence of two major peptide exchange pathways, one offering spontaneous release, the other one relying on DM catalysis. For the catalysed exchange pathway we reveal a series of spontaneously occurring conformational changes, which share some, but not all features with the DM-bound structure, and which potentially promote DM binding. Which of these pathways predominates and thus how immune responses are shaped is determined by an individual's particular combination of expressed MHCII alleles and available peptides.

## Results

### A stable pMHCII shows inherent conformational plasticity

**Figure 1:**
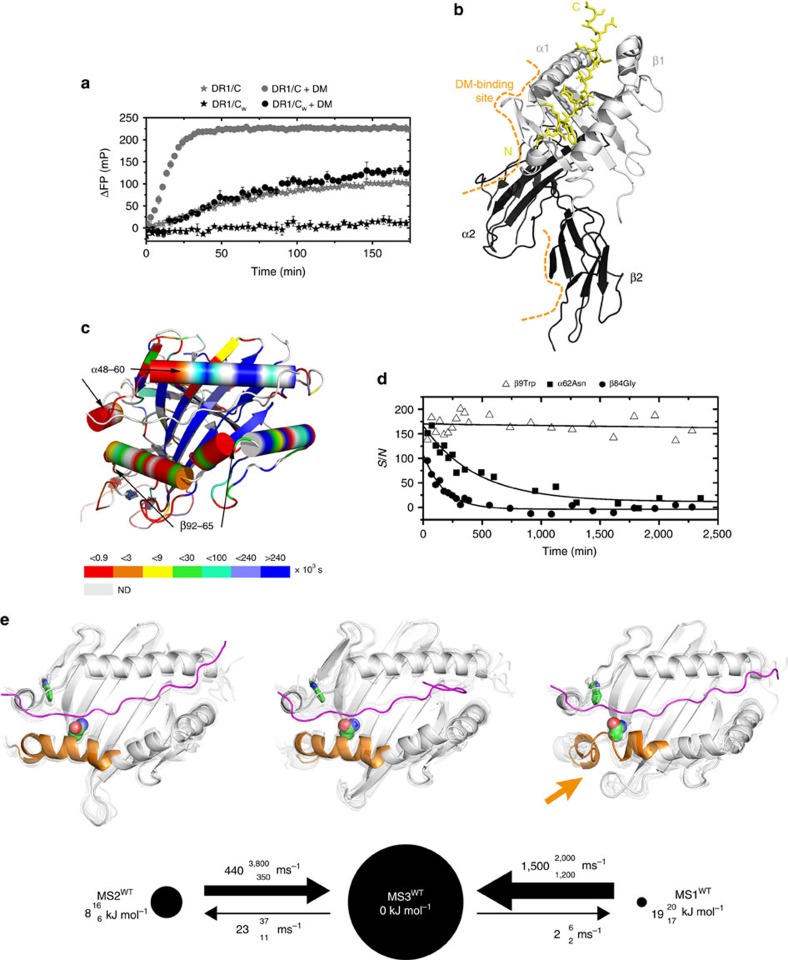
Conformational plasticity of an intrinsically stable and DM-unresponsive pMHCII complex. (**a**) Exchange of DR1-bound CLIP (C) or CLIP_M107W_ (C_W_) for HA_306-318_-FITC in the absence or presence of DM at 37 °C as detected by fluorescence polarization, performed as triplicates and representative of three independent experiments. (**b**) Crystal structure of DR1/C_w_. The DM-binding interface is indicated, DR1 α1/β1 and α2/β2 domains are coloured in light and dark grey, respectively. The peptide is shown in yellow with its N and C terminus marked. (**c**) Backbone HDX half-lives observed for DR1/C_W_ plotted onto the structure of DR1/C_W_, viewed from the top. ND means not determined. (**d**) Signal decay due to HDX plotted over time for selected α and β residues in DR1/C_W_. (**e**) Kinetic maps of Markov state models obtained from our in total 90 μs molecular dynamics simulations of DR1/C_W_. Metastable states (MS) are represented as black discs with areas proportional to the relative stationary weight of an MS. The black arrows in these maps indicate transitions between metastable sates, where the width of each arrow increases with the corresponding transition rate. Relative free energies and transition rates for the MSs are each presented with a ‘lower-/upper-case' 1*σ* confidence interval. Each MS is illustrated by ribbon representations of MHCII (white) and CLIP (magenta) of eight simulated conformations (one opaque, seven transparent viewed from the top into the binding groove). The α-helix of βA74-R93 is highlighted in orange, and the side-chains of αW43 and βN82/A as sticks and a space fill model, respectively. An orange arrow highlights the distortion of in the β-chain α-helix (β74-93). The simulated conformations were superimposed using the iterative fit VMD plugin^78^,^80^.

In order to unravel the impact of conformational plasticity on peptide exchange, we first characterized a kinetically stable pMHCII as reference, an HLA-DR1/CLIP complex with the P_1_-methionine of the natural placeholder CLIP_102–120_ substituted for tryptophan (DR1/CLIP_102–120_M107W, shortened: DR1/C_W_). The expected advantage of choosing this reference is an increased stability of the pMHCII as the tryptophan side-chain optimally fills the P_1_ pocket that dominates the DR1-binding groove, but cannot be accommodated by P_9_ and thus prevents peptide reorientation^20^. Indeed, the DR1/C_w_ complex is markedly more resistant to exchange against a fluorescein-labelled haemagglutinin (HA)-derived peptide than DR1 loaded with wild-type CLIP (DR1/C), as detected by an FP-based peptide-exchange assay (Fig. 1a). A small but significant increase of the stability of this complex is also reflected by a gain in midpoint denaturation temperature of approximately 4 °C (Supplementary Table 1). Additionally, we solved the crystal structure of DR1/C_W_ at 1.4 Å resolution (Fig. 1b and Supplementary Table 2), and confirmed that DR1/C_W_ has the standard canonical fold and that the new tryptophan side-chain is well accommodated by the P_1_ pocket.

Previous studies suggested that in order to be amenable for peptide exchange, a pMHCII must be able to transiently sample exchange-competent excited conformational states^13^,^14^,^21^,^22^,^23^. In order to identify secondary structure elements that could undergo conformational changes in solution, we subjected the DR1/C_w_ complex to NMR-detected H/D exchange (HDX) measurements that report the dynamical exposure of amide protons to the solvent. HDX protection factors were derived from exponentially-fitted (EX2 regime) intensity decays of backbone amide signals in a series of heteronuclear single quantum coherence (HSQC) spectra (Fig. 1c,d, Supplementary Table 3, Supplementary Fig. 1a,b). The results reveal that the N-terminal half (region coloured red in Fig. 1c) of the peptide-binding groove extending from the P_1_ to P_6_ pocket region, including residues αF48-L60 and βK65-Q92, displays strikingly little protection from HDX in DR1/C_w_. Additionally, the indole proton of the highly conserved αW43, which is needed to flip out of the peptide-binding groove to form an essential part of the DM interface^14^, is not protected from HDX indicating side-chain mobility.

Thus, although the crystal structure displays several hydrogen bonds in the P_1_ pocket region of the peptide-binding groove, this part of the protein appears to represent a rather flexible sub-module surrounding the N-terminal part of the peptide (Fig. 1c). This includes the side-chain of αW43 and the adjacent helical segments of both MHCII chains, thereby covering part of the main binding site of the exchange catalyst DM (see Fig. 1b for a sketch of the DM interface).

In order to reveal structural details and transition rates of the dynamical changes, we conducted extensive all-atom explicit-solvent molecular dynamics (MD) simulations. Over 160 simulation trajectories adding up to about 90 microseconds of total simulation time were collected—and thus two to three orders of magnitude more sampling than in previous simulation studies^9^,^10^,^11^,^12^—combined and analysed by a Markov state model (MSM)^24^,^25^ using the PyEMMA program^26^ (see Methods). In agreement with the HDX results, we found that several secondary structural elements could undergo spontaneous conformational fluctuations. Figure 1e shows the three most long-lived metastable states from our analysis that can interconvert on timescales of a few microseconds: The most stable ground state MS3^WT^, and the less stable excited state MS2^WT^ have an overall structured fold. Of special interest is MS1^WT^, a very low-populated state with a free energy of 19 kJ mol^−1^ above the ground state that exhibits unfolding of β1 α-helix residues C79-R93 (Fig. 1e, orange arrow, and Supplementary Fig. 2), consistent with the HDX results. Finally, we find that in all three simulated metastable states, there are spontaneous transitions of αW43 to the outward-flipped state (Supplementary Fig. 3), consistent with a side-chain conformation that resembles the DM-bound state^14^. Moreover, all three states exhibit flexible termini of the CLIP ligand, which may be an indication of early steps in a ligand-exchange pathway.

Taken together, the kinetically stable pMHCII DR1/C_W_ predominantly exists in a ground state, but the N-terminal end of the peptide-binding groove including the DM-binding site in the α1-domain as well as the β1-domain α-helix spontaneously samples low-populated conformations. Despite a well-occupied P_1_ pocket the outward-flipping of the αW43 side-chain appears to be a rare feature of both the ground state and the low-populated conformations.

### Indication of a rare conformation during peptide exchange

**Figure 2:**
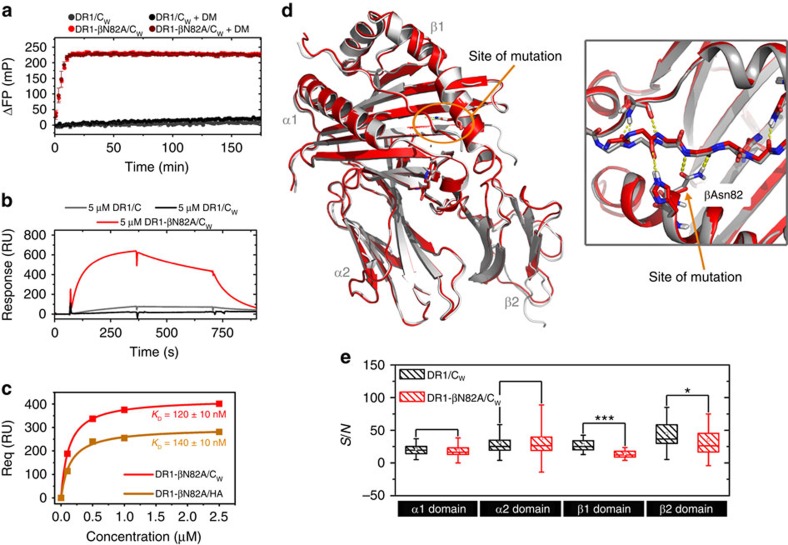
The intermediate state behaviour of DR1-βN82A/C_W_is not mediated by structural alteration. (**a**) Exchange of DR1- or DR1-βN82A-bound CLIP_M107W_ (C_w_) for HA_306-318_-FITC in the absence or presence of DM at 25 °C, performed as triplicates and representative of two independent experiments. Plotted are the mean values±standard deviation of the change in fluorescence polarization (ΔFP, calculated by subtracting the detected FP values of a protein-free reaction [HA-FITC only] from the reaction containing the DR/peptide complexes) over time. (**b**) SPR-detected binding of DR1/peptide complexes to immobilized DM. In all, 5 μM DR1/peptide complexes (DR1/C [wt CLIP] or DR1/C_W_ CLIP_M107W_ and DR1-βN82A/C_W_ CLIP_M107W_) are allowed to bind to the chip, washed with buffer and released by HA-peptide injection. (**c**) Req-plots for the SPR-detected binding of DM to DR1-βN82A bound to C_w_ or HA_306-318_ at different substrate (DR/peptide) concentrations, with calculated *K*_D_ values ± standard deviation resulting from two experiments. (**d**) Left: Structural alignment of DR1/C_W_ (grey) and DR1-βN82A/C_W_ (red). Right: magnification of the N-terminal-binding groove highlighting the H-bonds formed between βN82 and the P2 site of the peptide. (**e**) Box-and-whisker plot of signal-to-noise ratios (*S/N*) for backbone NH groups of the individual domains of DR1/CW and DR1-βN82A/CW (α1: assigned residues of 2-85, α2: 86-189, β1: 6-95 and β2: 96-193) showing the significant global increase in line broadening of the β1 domain in DR1-βN82A/C_W_ relative to the WT complex. The box includes the median and the 25^th^ and 75^th^ percentiles, respectively. The whisker represent 1.5 standard deviations. ***P<0.001, *P<0.05 (*t* test).

Interestingly, the flexible N-terminal end of the peptide-binding groove covers part of the main binding site of the exchange catalyst DM, spanning α1-chain residues αK39-αL60 (see Fig. 1b for a sketch of the DM interface). Moreover, a region on the opposite side of the peptide-binding groove (βV85-V91) has also been shown to rearrange upon DM binding^14^. Thus, it appears likely that enhanced dynamics of the N-terminal end of the peptide-binding groove are a prerequisite for DM interaction. Conformational plasticity of the DM-binding site in the α1 domain has already been suggested to represent a prerequisite for catalysed peptide exchange, and various mutations within this region have been shown to modulate DM susceptibility^13^,^14^,^21^,^27^. In order to unravel the relevance of the observed transient local unfolding of the β1 domain α-helix for peptide exchange, we asked for the structural and dynamic impact of a mutation that further destabilizes that region. We introduced an alanine in position β82 deleting the two H-bonds between βN82 and the peptide's P_2_ site, which have been shown to contribute remarkably to pMHCII stability^1^,^3^,^21^. Indeed, DR1-βN82A/C_W_ is a kinetically destabilized complex that displays a clear reduction in thermal stability of 19 °C (Supplementary Table 1), concomitant with a dramatic acceleration of the non-catalysed (DM-independent) peptide-exchange rate (Fig. 2a). Strikingly, Surface plasmon resonance (SPR) measurements report that the binding of the mutant to DM is massively increased, showing a *K*_D_ value in the 100 nM range for DR1-βN82A loaded with C_W_ as well as another high-affinity peptide (HA), while binding of DR1/C_W_ to DM was hardly detectable (Fig. 2b,c)^28^. Thus, DR1-βN82A/C_W_ captures features of a late intermediate of the peptide-exchange mechanism, as it tightly binds the catalyst without depending on its action.

In order to elucidate the basis of this phenotype, we solved the crystal structure of DR1-βN82A/C_W_. The limited resolution of 3.2 Å (Supplementary Table 2) was conceivably due to the reduced thermodynamic stability of this complex. Surprisingly, the DR1-βN82A/C_W_ structure was nearly indistinguishable from the high resolution DR1/C_W_ structure, with an root-mean-square deviation (RMSD) value of only 0.432 Å (Fig. 2d and Supplementary Fig. 4a). Also the comparison of B-factor distributions, normalized to the mean B factor of each chain, did not reveal gross differences between both structures with variability found in those regions that also differ between all HLA-DR1-CLIP structures deposited to date (data not shown). Moreover, nuclear Overhauser enhancement spectroscopy (NOESY) spectra of DR1-βN82A/C_W_ in solution did not indicate structural alterations if compared with the much more stable DR1/C_W_ complex (Supplementary Fig. 4b–d), and chemical shift differences between ^1^H-^15^N-TROSY-HSQC spectra of both DR1-βN82A/C_W_ and DR1/C_W_ were restricted to the site of mutation (Supplementary Fig. 5a,b). Having no indication for major structural changes in the peptide-bound conformation, we also ruled out that DR1-βN82A has lost most of the bound peptide and might behave as an empty MHCII: Spectra of DR1-βN82A/C_W_ are consistent with a peptide-loaded conformation as they did not show the massive loss of signals characterizing a destabilized binding groove in peptide-free DR1 (exemplified in Supplementary Fig. 5c), and the presence of peptide-loaded DR1-βN82A was further confirmed by peptide dissociation experiments (Supplementary Fig. 5d–f).

Thus, even in the context of an optimal P_1_ anchor residue, a mutation in the β1 domain α-helix leads to a kinetically instable complex (DR1-βN82A/C_W_) that tightly binds DM. This biochemical phenotype is not explained by the ground-state structure as detectable by crystallography and NMR.

### HLA-DR1 excited states rationalize catalysed exchange

As the biochemical phenotype of DR1-βN82A/C_W_ could not be explained by its ground-state structure, we wondered whether its structural ensemble comprises excited metastable states that account for accelerated peptide-exchange and increased DM binding.

A first indication for a difference in conformational plasticity between DR1-βN82A/C_W_ and DR1/C_W_ is given by a comparison of ^1^H-^15^N TROSY-HSQC peak intensities: Signal-to-noise levels of the DR1-βN82A/C_W_ β1 domain are reduced to 48% as compared with DR1/C_w_ (Fig. 2e), suggesting a higher plasticity of DR1-βN82A/C_W_ compared with DR1/C_W_. This effect remained significant at lower temperatures (Supplementary Fig. 6).

**Figure 3:**
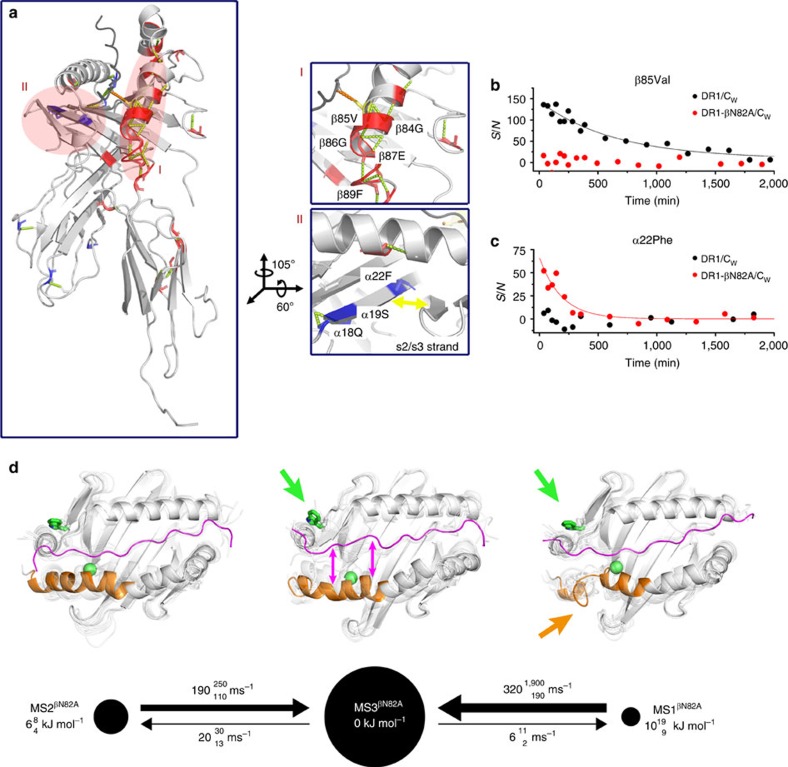
DR1-βN82A/C_W_ transiently features DM-susceptible conformations. (**a**) DR1-βN82A/C_W_ backbone NH HDX rates significantly enhanced (red) or reduced (blue) as compared with DR1/C_W_ are highlighted in the DR1/C_W_ crystal structure, with magnification of two different regions. (**b**,**c**) Comparison of the change in *S/N* between DR1/C_W_ and DR1-βN82A/C_W_ upon HDX for the NH groups of β85Val (**b**), located in region I, and α22Phe (**c**) located in region II . (**d**) Kinetic maps of our MD simulations of DR1-βN82A/C_W_ with metastable sates, transition rates and conformations represented as in Fig. 1g. Coloured arrows highlight characteristic conformational changes, that is, the α-helix distortion of β74-93 (orange) the side-chain flip of αTrp43 (green), and partial dissociation of CLIP (magenta).

In order to give a detailed account of these altered dynamics we performed HDX measurements as described above and observed greatly amplified internal dynamics in DR1-βN82A/C_W_ compared with DR1/C_W_ (Fig. 3a–c and Supplementary Table 4). In particular, the β1 domain α-helix residue βR80 and region βG84-R93 in the vicinity of the mutation were destabilized (Fig. 3a, region I).

The impact of these increased internal dynamics on the conformational space of DR1-βN82A/C_W_ was again analysed using a total of 90 microseconds of explicit-solvent MD simulations and a subsequent Markov model analysis (Fig. 3d). These simulation data show a qualitatively similar picture as for DR1/C_W_, but with shifted populations and enhanced structural flexibility. The ground-state MS3^βN82A^ has a mostly stable fold similar to DR1/C_W_, in agreement with our X-ray structures, NOESY measurements and chemical shift perturbation data. Again, two excited metastable states with low populations are found, one with a well-defined fold (MS2^βN82A^), and one where the β1 domain α-helix is largely unfolded at residues βQ70-R93 (MS1^βN82A^, Fig. 3d, orange arrow and Supplementary Fig. 2). The α-helix unfolding is more pronounced than the partial helix breaks observed in DR1/C_W_. However, the most striking effect is the relative stabilization of MS1^βN82A^ over MS1^WT^ by 9 kJ mol^−1^ (from 19 kJ mol^−1^ to 10 kJ mol^−1^—compare Fig. 3d and 1e). While it is still a low-populated metastable state with a population fraction of about 1.6% and has thus escaped characterization by X-ray and NMR measurements, its population is now increased by a factor of 28.

Several features of this excited metastable state suggest that MS1^βN82A^ represents an intermediate state along the peptide exchange pathway that can be spontaneously sampled in timescales of about 50–100 microseconds: The β1 domain α-helix conformation in MS1^βN82A^ is in good agreement with the partially unfolded helix in the DR1/DM complex structure^14^. Additionally, the optimal arrangement of the DM-binding site goes along with an alignment of α-chain β-strands s2 and s3 (Fig. 3a, region II) leading to novel interstrand H-bonds in DM-bound structures^14^,^29^. The increased protection of the α22F amide observed in HDX experiments of DR1-βN82A/C_W_ (Fig. 3a,c) is in agreement with an on average more proximal orientation of the two strands in the mutant. An increased protection of the adjacent potential H-bond partners α20G/α36M is not observed, indicating that a full line up of s2 and s3 strands, as seen in the DM/DR1 crystal structure, might rather occur by an induced-fit mechanism. Instead, increased protection of α18Q and α19S amide protons additionally indicate stabilized H-bonds between strands s3 and s4 in DR1-βN82A/C_W_ and our simulation results indicate a stabilization of the helical residues αE55-Y79 on top of these β-strands in MS1^βN82A^ as compared with MS3^WT^ (Supplementary Fig. 7). Overall, however, the entire region around the N terminus of the peptide displays a notable increase in mobility (Supplementary Fig. 7). This increased mobility likely facilitates the conformational change of the αW43 side-chain to flip outward and thus to participate in the DM-binding interface, as seen in the DR1/DM crystal structure^14^. As α43W Hɛ chemical shift differences between wild-type and mutant pMHCII are relatively small in ^1^H-^15^N TROSY-HSQC spectra (Supplementary Figs 4e and 5b), it has to be concluded that the flipped-in orientation of the α43W side-chain is still dominant in DR1-βN82A/C_W_. This is confirmed by our MD simulation results, however, we find the probability of being in a flipped-out state has more than doubled in the DR1-βN82A/C_W_ mutant compared with DR1/C_W_ (that is, from 0.063±0.003 to 0.16±0.02) (Supplementary Fig. 3). In order to account for this population increase, MS1^βN82A^ and MS3^βN82A^ are represented by conformers which show the low-populated flipped-out α43W side-chain conformation in Fig. 3d (green arrows).

Taken together, the βN82A mutation destabilizes the β1 domain α-helix fold, which contributes to an overall increase of the mobility in the entire region surrounding the peptide N terminus. This destabilization shifts the conformational ensemble towards the low-populated state that shares features of the DM-bound conformation^14^ and thus rationalizes the phenotype of DR1-βN82A/C_W_ in the DM-catalysed peptide exchange pathway.

### Non-catalysed peptide exchange pathway

A key feature of DR1-βN82A/C_W_ is its high and DM-independent peptide-exchange rate (Fig. 2a), which cannot be explained by the simulated excited state MS1^βN82A^ but MS3^βN82A^:

Compared with DR1/C_W_, in DR1-βN82A/C_W_ we observe the peptide's residues S105, K106, R108-A110, P112 and A117 to have a significantly lower contact frequency to any DR1 residue (magenta arrows in Fig. 3d, Supplementary Fig. 8a). This is associated with a significantly higher frequency of most of these residues (S105-R108, A110) to interact with water (Supplementary Fig. 8b). Interestingly, most of these partial CLIP dissociation events occur more often in MS3^βN82A^ than in MS1^βN82A^ (Fig. 3d, Supplementary Fig. 8a).

Thus, our hypothesis is that two major distinct pathways for peptide exchange are likely to exist in parallel: while the kinetic stability of the ground state defines the propensity of an individual pMHCII for uncatalysed peptide exchange, the population of rare conformations determines DM susceptibility.

### Validations of the model of two major exchange pathways

As shown above, the equilibrium between the two peptide-exchange pathways exhibiting different DM responsiveness can be shifted by a single βN82A point mutation at the P_2_ site. In both our HDX measurements and MSM analyses of MD simulations (Fig. 3 and Supplementary Fig. 7), a characterizing feature of this shift is the unfolding of the MHCII β1 domain α-helix at residues βQ70-R93. The βN82A mutation, however, does not only modulate the β1 domain α-helix stability but also markedly affects peptide dissociation by the removal of two H-bonds.

**Figure 4:**
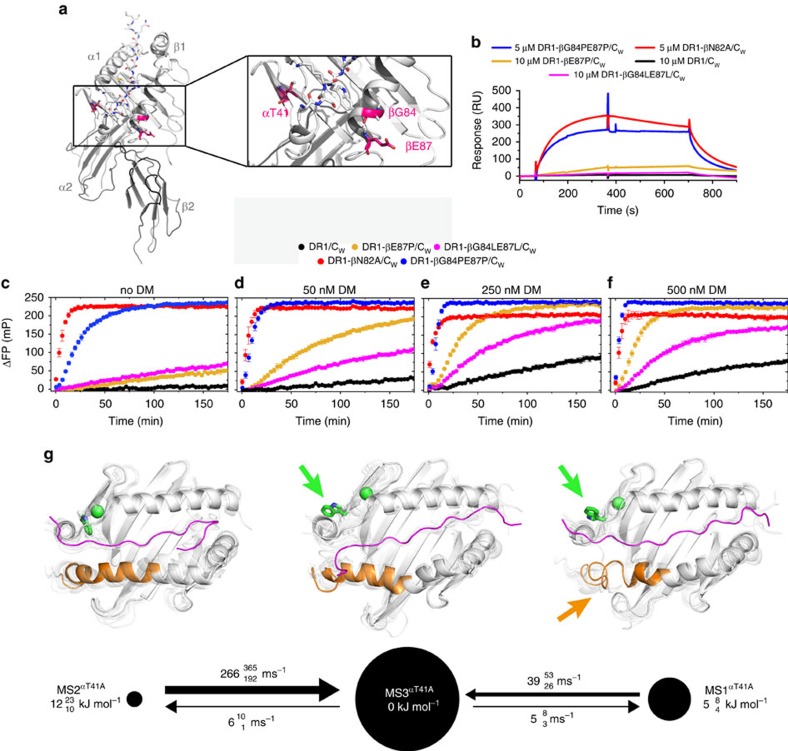
DM-susceptibility is associated with β1 domain α-helix unfolding. (**a**) Shown is the structure of DR1/C_W_ with the magnified β1domain α-helix. Two residues (β84G and β87E) that point away from the binding pocket were chosen to either destabilize (by substitution for proline) or stabilize (by substitution for leucine) the β1 domain α-helix. Additionally, a mutation (DR1-αT41A) on the opposite site of the binding groove previously described to influence DM susceptibility^27^ was analysed by simulation. (**b**) Binding of streptavidin-immobilized biotinylated DM to different DR1/peptide complexes. Measurements were performed as described in Fig. 2b and are representative of at least three experiments. (**c**–**f**) Exchange of DR1-bound C_W_ for HA_306-318_-FITC in dependence of DM as detected by fluorescence polarization. Experiments were performed as triplicates and are representative of at least two independent experiments. Plotted are the mean values±standard deviation of ΔFP over time. (**g**) Kinetic Maps of MD simulations of DR1-αT41A/C_W_ with metastable sates, transition rates and conformations represented as in Figs 1e and 3d.

Hypothesizing two independent exchange pathways, we anticipate that destabilizing the β1 domain α-helix without touching the H-bond network between MHCII and peptide should be sufficient to shift the conformational equilibrium towards the DM-susceptible state, while helix-stabilization should not enhance DM binding. In order to test this hypothesis, we introduced one or two helix-destabilizing proline residues into the β1 domain α-helix of the wild-type DR1 molecule at positions pointing away from the binding groove (DR1-βE87P and DR1-βG84PβE87P) and additionally aimed at stabilizing the helix by replacing the same residues by leucine, which has a higher propensity for helix formation due to hydrophobic contacts (DR1-βG84LβE87L) (Fig. 4a).

SPR measurements show that introduction of one proline (DR1-βE87P/C_W_) or two (DR1-βG84PβE87P/C_W_) leads to complexes with gradually increased binding to DM, whereas DR1-βG84LβE87L/C_W_ shows a similarly negligible response to DM as DR1/C_W_ (Fig. 4b). Strikingly, DR1-βE87P/C_W_'s non-catalysed peptide exchange is slightly increased as compared with DR1/C_W_, but clearly can be enhanced overproportionally by the addition of DM. For DR1-βG84PβE87P/C_W_, the non-catalysed peptide exchange is strongly enhanced but can be further accelerated upon addition of DM (Fig. 4c–f). In contrast to that, DR1-βG84LβE87L/C_W_ shows slightly increased intrinsic peptide exchange rates but DM does only improve exchange to a level comparable to DR1/C_W_. Thus, a successive destabilization of the β1 domain α-helix by the introduction of proline, especially when placed at position 87, shifts the conformational equilibrium from the DR1/C_W_ situation towards a more DM-susceptible conformation. To the contrary, the introduction of helix-stabilizing leucine residues does not significantly improve DM susceptibility.

To validate our model based on previous experimental design we capitalized on the mutant DR1-αT41A, that has been described to enhance DM binding and DM-dependent exchange rates without accelerating non-catalysed peptide dissociation of a high affinity peptide^27^. According to our model, this phenotype would be consistent with a stabilization of the DM-susceptible MS1 states. We tested this hypothesis with an aggregated total of 95 microseconds of explicit-solvent MD simulations and a subsequent Markov model analysis of DR1-αT41A/C_W_. Indeed, we observe a clear destabilization of the same region of the β1 domain α-helix in MS1^αT41A^ and MS2^αT41A^ as observed in MS1^βN82A^ and MS2^βN82A^ (Fig. 4g, orange arrow, Supplementary Figs 2 and 7), although the mutation is introduced on the opposite side of the peptide-binding groove. Despite the mutation's direct vicinity to the α43W side-chain, flipped-out conformations are similarly frequent in MS1^αT41A^ and MS3^αT41A^ as in MS1^βN82A^ and MS3^βN82A^, respectively (Fig. 4g, green arrows, Supplementary Fig. 3). Moreover, the burial of the CLIP peptide side-chains in DR1-αT41A/C_W_ is similar to DR1/C_W_, and thus the peptide is more tightly bound than in MS3^βN82A^ (Supplementary Fig. 8a). However, residue P_-1_ shows more frequent solvent encounters in MS3^αT41A^ (Supplementary Fig. 8b), thus arguing for flexibility to increase at the very N terminus of the peptide. All these observations go along with an even more pronounced stabilization of MS1^αT41A^ over MS1^WT^ by 14 kJ mol^−1^ (compared with 9 kJ mol^−1^ for MS1^βN82A^, see Supplementary Fig. 7), and provide a possible explanation for why in clear contrast to the βN82A mutation, αT41A does not significantly enhance non-catalysed exchange rates of a high affinity peptide^27^.

Thus, consistent with our model, the equilibrium between non-catalysed and DM-catalysed peptide exchange pathway can be shifted towards the latter by mutations that either directly target the stability of the β1 domain α-helix at residue β87 without removing H-bonds between MHCII and peptide, or by a mutation of a residue at the opposite side of the binding groove that is part of the DM interface.

## Discussion

Conformational plasticity has long been suspected to lie at the heart of recapitulating antigen exchange at the molecular level^13^,^21^,^27^,^30^,^31^,^32^,^33^. Comparing the wealth of solved MHCII-peptide structures hinted at structural variability that might well extend beyond the ground-state structures seen so far^18^,^19^,^22^. Two landmark studies by Pos *et al*.^14^ and Guce *et al*.^29^ unambiguously proved the MHCII fold to be prone to large structural rearrangements when complexes of either MHCII/DM or DM/DO form. The timescale and order of these transitions as well as their relevance for either catalysed or uncatalysed peptide exchange remained enigmatic.

**Figure 5:**
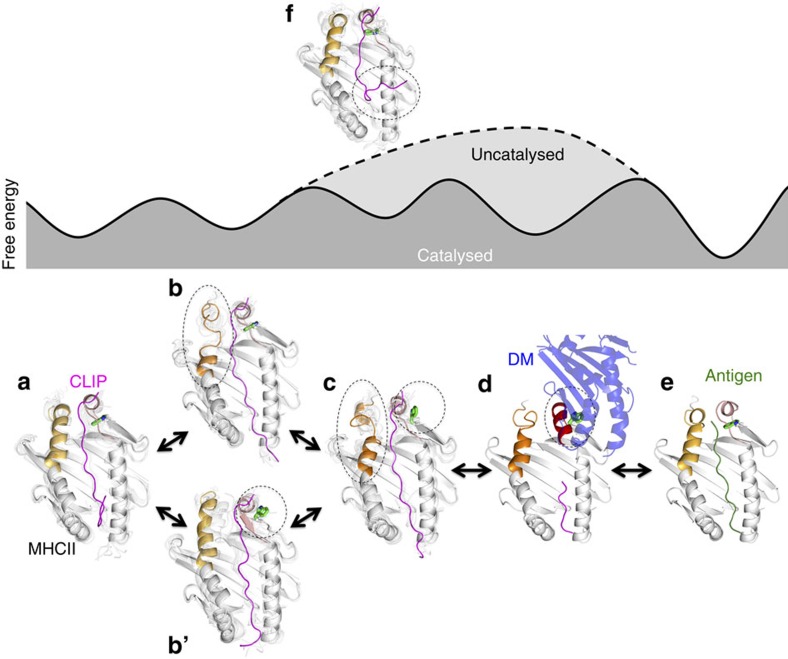
Thermodynamic and kinetic models for catalysed and non-catalysed MHCII-peptide exchange. Along these modelled pathways with sketched free energy surfaces, representative conformations are shown of MHCII (white ribbons, top view into the binding groove), CLIP (magenta), antigen (green, sketched) and DM (blue ribbons). In **a**, **b**, **b'**, **c**, **f**, we present simulated conformations of MS3^WT^, MS1^βN82A^, MS3^βN82A^, MS1^βN82A^ and MS3^βN82A^, respectively, **d** and **e**, crystal structures with PDB IDs 4FQX and 1DLH, respectively. In MHCII, helical residues of αR44-E55 and βA74-R93 are highlighted in red and orange, respectively, and the αW43 side-chain is highlighted in green. The dashed ellipses point out key conformational events along each peptide exchange pathway.

Here we combined NMR and computational MD approaches to compare the conformational plasticity of a stable pMHCII (DR1/C_w_) that is characterized by low affinity for DM, but exerts DM-dependent peptide exchange, with the kinetically destabilized mutant DR1-βN82A/C_w_. This mutant, in contrast, showed strong DM-binding albeit with DM-independent peptide exchange. Based on aggregate results we are now able to propose a detailed mechanistic model of MHCII peptide exchange (Fig. 5). The proposed pathways were rationalized by the comparison of model sub-states to the DM/DR complex (Supplementary Fig. 9) and validated by the analysis of further mutations (βE87P, βG84PβE87P, βG84LβE87L and αT41A):

The propensity for intrinsic, uncatalysed peptide exchange is determined by the kinetic stability of a pMHCII's ground state (Fig. 5a, Supplementary Fig. 9A, Supplementary Movie 1), which rarely samples slightly higher energy states that are characterized by flipping-out of the α43W side-chain and/or a destabilized β79-93 helix (MS1^WT^ in Fig. 1e and Supplementary Fig. 2). Destabilizing mutations such as βN82A or βG84PβE87P can lead to a ground state that is clearly more prone to intrinsic peptide exchange than in case of the stable complex DR1/C_w_.

The catalysed peptide exchange pathway, however, occurs from the MHCII ground-state structure (Fig. 5a) via multi-step transitions (Fig. 5b,b', Supplementary Movie (1,2,3)) towards an energetically excited intermediate (Fig. 5c) that exhibits features of a DM/DR complex^14^ (Fig. 5d) with the least number of significant polar interaction differences with respect to the DR1/DM crystal structure among all the simulated metastable states (Supplementary Fig. 9). Thus, the propensity for DM-catalysed peptide exchange depends on the sampling of a low-populated state where the βC79-R93 α-helix partially unfolds and the α43W side-chain is in the flipped-out conformation (Fig. 5c, Supplementary Fig. 9c). The resulting transient states that already carry features of the DM-bound conformation are spontaneously sampled on timescales of about a hundred microseconds. Mutations can shift the conformational equilibrium towards higher DM affinity, as observed for DR1-βN82A/C_w_ and DR1-αT41A/C_w_, in which the excited metastable state MS1^βN82A^ and MS1^αT41A^ is 28-fold and 200-fold, respectively, higher populated than in DR1/C_W_. DM would then select for conformations with unfolded βC79-R93, a flipped-out α43W and a flexible peptide N terminus (Fig. 5d, Supplementary Fig. 9d).

Interestingly, in the DM-bound^14^ form, the DR1 β-helix is still destabilized as evidenced by the distortion of βE87 away from the helical conformation. Two critical intra-helical hydrogen bonds provided by this residue are not seen in the DM-bound DR1 and when introducing a proline at this position the NH-based hydrogen bond to βY83(CO) is disrupted and nicely explains the increased DM susceptibility of this mutant in the framework of our model (Fig. 4). The αR44-E55 helix that is observed in the DR/DM complex^14^ (Fig. 5d, Supplementary Fig. 9d), is assumed to fold as part of an induced fit, as we do not detect its spontaneous formation, neither experimentally nor in our simulations. Finally, upon DM-binding, peptide exchange occurs and DM dissociates (Fig. 5e, Supplementary Fig. 9e).

We postulate that the equilibrium between the non-catalysed as well as the DM-catalysed exchange pathways can be skewed as a result of various modifications as implied by the investigated mutations on both sides of the binding groove. As peptide affinity influences kinetic stability of pMHCII complexes, binding groove pocket occupation will also affect the populations of the conformational states. In this study, we used a CLIP peptide variant modified to increase the stability of the pMHCII complex in order to derive features independent of the effect of non-optimal pocket occupation. In case of wild-type CLIP, which has a sub-optimal P_1_ pocket packing, we expect based on its reduced kinetic stability an increase of both non-catalysed and DM-catalysed peptide exchange. An empty P_1_ pocket^13^, however, does not appear to be the only prerequisite for the formation of DM-susceptible conformations, as indicated by the αT41A mutation^27^ (Fig. 4g). Thus, in the context of a low-affinity peptide we would anticipate that destabilizing mutations or polymorphisms have an additive effect and that enthalpic and entropic contributions from the entire pMHCII system determine the DM susceptibility of an individual pMHCII (refs 27, 33).

Our data are in agreement with previous observations that dynamics in the 3_10_-helical region of the DR α-chain and the P_1_ pocket are of great relevance for the peptide-exchange reaction^15^,^16^,^21^, but we provide evidence that the stability of the adjacent helical hinge segment in the β-chain α-helix is also important, as its loss in DR1-βN82A/C_W_ is associated with enhanced peptide-exchange rates (Figs 1, 3 and 4, Supplementary Figs 2 and 7, Supplementary Table 4). This is supported by the observation that the DM/DR crystal structure and MD simulations of the peptide-free conformation of DR show structural re-arrangements involving MHCII residues βV85-V91 and βC79-Y83, respectively^10^,^14^. Taking into account that transient helix unfolding as observed for DR1 β-chain residues βC79-R93 is a relatively slow event, it appears conceivable that MHCII β-chain dynamics represent a major step to free the system and provide an entropic contribution to arrange the DM interface located in the α-chain. Communication across the peptide-binding groove is confirmed by our simulation of DR1-αT41A/C_W_, where a mutation in the α1 domain indirectly influences the stability of the β1 domain α-helix (Fig. 4a,g). A previously described hydrophobic network (β89F, β153W, α48F)^21^, could function as a central allosteric communication switch to translate dynamic modulation across the binding groove.

Furthermore, we anticipate that our model can help to explain the variability in DM susceptibility^13^,^15^,^16^,^34^ and peptide exchange rates observed for DR and DQ alleles. Interestingly, the low DM susceptibility observed for several DQ alleles^15^,^16^,^35^ that represent risk factors for autoimmune disorders goes along with the incorporation of residues with a higher helical propensity in the region βG84-T90 and several additional hydrogen bonds lead to an extension of the DR1 helical segment βR80-R90 to residue βR93. Thus, these DQ alleles, namely DQ2 and DQ8, show a stabilization of a structural segment, which we here prove to be a highly sensitive element of conformational plasticity, and we argue that the dynamic profile in these DQ alleles is modulated in a way that conformations lying on-pathway for peptide exchange and DM binding are less frequent, due to an increase in β-chain α-helix stability. On the other hand, alleles DQ1 and DQ6, which are protective with regard to T1D and which display enhanced DM susceptibility, show a more open and thus less stable helical segment βG84-T90, a feature that might be coupled to altered 3_10_ helical flexibility for these variants.

We see our investigation as a contribution towards a unifying concept of antigen presentation by class II molecules. Clearly, the two major pathways described in this study are not strictly to separate since most mutants or polymorphic variants will affect both scenarios. On the one hand, changing the pocket occupation or hydrogen bond network between MHCII and peptide will directly impact intrinsic peptide release rates and increased peptide dissociation will propagate to the flexible helical elements required for DM binding. On the other hand, mutations more remote from the binding groove such as the αT41A or βE87P DR1 variants also investigated here are well suited to reveal the more peptide-independent structural elements required for DM editing and pave the way to understand the impact of certain polymorphisms as discussed for the DQ alleles.

Furthermore, our model could help to explain the action of small molecules on MHCII peptide exchange: several described compounds appear to act at the N-terminal end of the peptide-binding groove^36^,^37^,^38^,^39^, but direct experimental approaches to confirm their binding sites have as yet remained fruitless. We postulate that small molecule loading enhancers act on low-populated conformational states described in this study. Taking that idea further, drugs that induce adverse reactions based on their interference with antigen presentation as shown for Abacavir and MHCI^40^,^41^ might not only alter the presented peptide repertoire by modifying pocket properties, but also by influencing the exchange pathway.

The above presented concept of the relevance of the conformational landscape of a ‘receptor' molecule on downstream pathways might not only be applicable to the closely related tapasin-catalysed peptide loading of MHC class I molecules^42^,^43^,^44^,^45^ but is also known to other biological systems: The energy landscape of G-protein coupled receptors that strongly impacts activation pathways has been shown to depend on the individual receptor/ligand complex^46^ and as shown in this study, ligands might not act on the crystallizable ground states but on low-populated excited states^47^. For example, an order-to-disorder transition of two helical segments in the rhodopsin effector Gαi1 has been postulated and mutations that affect the thermodynamic stability of this region were shown to have a large effect on GDP release^48^. To determine the existence and rate of formation of the implied rare conformations will be an important step in a more universal understanding of G-protein coupled receptor activation.

In summary, we are able to rationalize key aspects of MHC class II dependent antigen binding and exchange. Our work thereby provides a blueprint for a thorough understanding of the complex interplay between allelic variance and peptide repertoires as they ultimately form and trigger T helper cell responses in wanted and unwanted immune responses.

## Methods

### Expression and purification of soluble MHCII proteins

DRA*0101 and DRB1*0101 (residues 1-192 and 1-198, respectively^49^) subunit-derived constructs were generated by site-directed mutagenesis (SDM) using the standard QuickChange protocol to introduce mutations. For crystallization of DR1-βN82A, a CLIP_106-120M107W_ peptide was genetically linked via a flexible tri-G_4_S-linker to the N terminus of the β-subunit^50^,^51^.

HLA-DR proteins were produced as described previously^49^,^52^. To generate pMHCII complexes, empty MHCII molecules were loaded with a 20-fold molar excess of peptide in the presence of 2 mM Ac-FR-NH_2_ dipeptide (loading enhancer) for 48 h at 37 °C. Soluble monomeric pMHCII complexes and peptide-free MHCIIs were finally obtained by applying the proteins and loading reactions onto a size exclusion chromatography column and buffer exchanged to PBS, pH 5.8. DM was expressed and purified using the baculovirus-insect cell expression system (pFastBacDual-Sf9) as previously described^28^.

### Fluorescence polarization assays

Loading of 200 nM HA_306-318_-fluorescein isothiocyanate (HA-FITC) on 1 μM DR1/peptide complexes was followed by fluorescence polarization in the absence or presence of 250 nM DM (if not stated otherwise). The reactions were set up in 40 μl in triplicates in PBS buffer, pH 5.8 at 37 °C (Figs 1a and 4b). In order to resolve loading of DR1-βN82A/C_W_ experiments were performed at 25 °C (Fig. 3a). Peptide dissociation of 150 nM DR/HA_306-318_-FITC complexes was monitored in the presence and absence of 20 μM competitor peptide (HA_306-318_) and 150 nM DM in PB/Citrate buffer, pH 5.5 at 37 °C as previously described^28^. ΔFP values were calculated by subtracting the measured fluorescence polarization of a protein-free reaction (HA-FITC only) from the reaction containing the DR/peptide complexes.

### Surface plasmon resonance

In all, 400–500 RU of biotinylated DM was coupled to streptavidin (SAD 500, Xantec bioanalytics, Germany), as previously described^13^. Experiments were carried out at 30 °C in 50 mM citrate buffer, 150 mM NaCl, pH 5.35, 0.06% C12E9 detergent, with a flow rate of 15 μl min^−1^ in a Biacore 3,000 device (GE Healthcare). Non-specific binding of the reference flow cells was subtracted from the DM-coupled flow cells. DR1/peptide complexes were injected for 300 s into DM-immobilized or control flow cells followed by 300 s buffer injection and 450 s of 50 μM HA peptide. The chip was regenerated by injecting high-affinity peptides for the respective HLA-DR alleles in the flow cell at concentrations ranging from 20 to 100 μM.

### Thermofluor assay

In order to determine the thermal stability, 0.2–0.5 mg ml^−1^ monomeric pMHCII protein was mixed with 5 × Sypro Orange (Life Technology). While the temperature was increased for 2 °C min^−1^, the emission was detected at 575 nm in lifetime after exciting the dye at 490 nm. Fluorescence intensity was plotted versus the temperature and a sigmoidal function was fitted to determine the midpoint temperature of the unfolding reaction (*T*_m_). Stability measurements were performed in PBS buffer, pH 5.8.

### Nuclear magnetic resonance

NMR spectra were acquired on a Bruker AV700 MHz spectrometer equipped with a 5 mm triple-resonance cryoprobe. Backbone assignments of approximately 90% α- and around 80% β-chain resonances were obtained for DR1/C_w_ and DR1-βN82A/C_W_ by standard three-dimensional experiments in combination with transferring assignments from DR1/C spectra^20^.

All NMR measurements were performed at 310 K in PBS buffer pH 5.8 containing 10% D_2_O with 35–100 μM pMHCII. Spectra were processed with Topspin (Bruker) and analysed with CcpNmr Analysis^53^.

MHCII complexes were partly deuterated α and fully deuterated β and ^15^Nα- and/or ^15^Nβ-labelled. DR1-βN82A samples used for assignments were additionally ^13^C labelled. NOESY-HSQC spectra with 80 ms mixing time were acquired for ^2^H- ^15^Nα/β-labelled DR1-βN82A/C_w_ with specific labelling of ^1^H-^15^N-Ala/Trp in the α and ^1^H-^15^N- Trp in the β-chain.

Chemical shift perturbation was calculated according to equation 1:


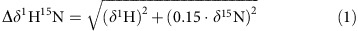


CcpNMR Analysis was used to determine the average spectral noise levels from peak-free regions. For signal to noise level calculation peak-heights were determined from ^1^H-^15^N-TROSY-HSQC spectra of 50 μM ^15^N β-labelled DR1/C_w_ (144 scans, noise level 2,450), 50 μM DR1-βN82A/C_w_ (144 scans, noise level 2,594), 60 μM ^15^N α-labelled DR1/C_w_ (144 scans, noise level of 5,093) and 35 μM ^15^N α-labelled DR1/C_w_ (340 scans to account for the difference in protein concentration, noise level of 3,997) measured at 37 °C for Fig. 2e.

### H/D exchange

Protected hydrogen atoms can exchange against deuterium in a transient energy state of a protein region, typically involving H-bond breakage 54. For native state proteins at pH values below 10, the EX2 limit can be used to obtain the equilibrium constant for the exchange according to equation 2:


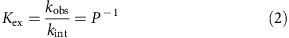


where *k*_obs_ is the observed exchange rate, *k*_int_ the intrinsic exchange, which has to be experimentally determined (or derived from the Sphere databases, http://landing.foxchase.org/research/labs/roder/sphere/), and P^−1^ is the amide protection factor. Based on the knowledge of *K*_ex_, the energy difference between the open and closed state is calculated using the following equation:


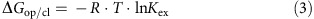


where Δ*G*_op/cl_ refers to the energy difference between the open and the closed state, *R* to the universal gas constant (0.00831462, kJ K^−1^ mol^−1^) and *T* to the absolute temperature in Kelvin.

Partly deuterated α and fully deuterated β ^15^Nα- and/or ^15^Nβ-labelled DR1/C_W_ complexes were concentrated to 80–100 μM in PBS buffer, pH 5.7, 10% D_2_O, 0.02% NaN_3_. Reference HSQC spectra were acquired with 1,024 × 96 complex data points and 16 scans at 37 °C. Afterwards, these DR1/peptide complexes were shock frozen and lyophilized overnight. Immediately after re-solubilization in 100% D_2_O a set of 90 HSQC experiments were acquired with a measurement time of ∼35 min per HSQC. At least 20 data points of the HSQC data set was used to plot *S/N* ratios of NH peaks against the experiment time. NH groups exchanging to an undetectable level within the dead time of the HSQC measurement (∼35 min) were considered to have half-lives (*t*_1/2_) of <15 min, while NH groups not showing adequate signal decay after 60 h were classified as highly stable, with a *t*_1/2_ >4,000 min and peaks with overlapping maxima were mostly excluded from the analysis. For peaks, which showed a clear decay within the experiment time (∼60 h), an exponential decay function of first order was fitted to the data according to the EX2-limit^55^. *K*_ex_^−*1*^ was obtained by normalizing the observed exchange rates for the intrinsic exchange rates (an average for the intrinsic rates was calculated with respect to the i±1 position; http://landing.foxchase.org/research/labs/roder/sphere/).

All together, 99 DR1/C_W_ and 124 DR1-βN82A/C_W_ residues displayed *t*_1/2_ of <15 min, 87 DR1/C_W_ and 72 DR1-βN82A/C_W_ residues displayed *t*_1/2_ >4,000 min, 85 DR1/C_W_ and 64 DR1-βN82A/C_W_ residues could be fitted.

### Crystallization and structure determination

Both proteins were crystallized using commercial sparse matrix screens. DR1 was purified and refolded from *Escherichia coli*. To obtain DR1/C_W_ the peptide was loaded a posteriori using a 10- to 20-fold molar excess of peptide in the presence of 1 mM AdEtOH^37^ or 2 mM Ac-FR-NH_2_ (ref. 39) as molecular loading enhancer overnight at 37 °C. Unbound peptide was removed by size exclusion chromatography using a Superdex 200 column (GE Healthcare, USA) into 20 mM MES, 50 mM NaCl, pH 6.4. The complex was concentrated to 10 mg ml^−1^ and subjected to crystallization by vapour diffusion in sitting drops. Diffraction-quality crystals grew within a few days in 0.2 M K/Na-tartrate, 20% (w/v) PEG3350, 0.1 M BisTrisPropane, pH 8.5.

For DR1-βN82A/C_W_, the protein was purified and refolded from *E. coli* and gel filtrated using a Superdex 200 column equilibrated in 20 mM MES, 50 mM NaCl, pH 6.4. Protein was concentrated and mixed with an equimolar amount of DM purified from S2 cells in the same buffer. The final protein concentration was 10 mg ml^−1^. Crystals were grown by vapour diffusion in sitting drops in 0.2 M Na nitrate, 20% (w/v) PEG 3350, 0.1 M BisTrisPropane, pH 7.5.

In both cases, crystals were harvested and flash frozen in the crystallization condition supplemented with 20% glycerol. Data from these crystals were collected at beamline 14.1 at Helmholtz-Zentrum Berlin.

In both cases, data sets were processed using the XDS package^56^. Initial phases were obtained by molecular replacement using the program PHASER^57^ using HLA-DR1 (PDB 2G9H) as a search model with the coordinates of the peptide removed. The final models were built using iterative rounds of refinement with the Phenix program package^58^ interspersed with manual model building in COOT^59^.

Despite the presence of equimolar amounts of DM in the crystallization conditions for the crystals of DR1-βN82A/C_W_, no DM could be identified in the electron-density. Both structures were deposited at the Protein Data Bank with accession codes 4X5W and 4X5X.

### MD set-up

The DR1/C (3PDO) and DR1/C_W_ (4X5W) crystal structures were analysed. pKa values of titratable residues for each structure were estimated with the program propKa^60^. Together with the gromacs program pdb2gmx^61^ and previous MD simulation studies performed on MHCII (ref. 12), protonation states were assigned for MHCII-physiological pH=5.8 that are consistent among these structures (fully-protonated His: α5/33/177, β16/81/111; ɛ-prot. His: α143/149/167, β112/177, prot. Asp: α25/29, prot. Glu: α11/21), together with disulphide bridges we introduced (αC107-αC163, β15-β79, β117-β173). In DR1, unresolved atom coordinates of αL105-F112, βQ107-H112 were modelled by superposition with corresponding templates taken from 3PDO. Standard (charged) termini were used for chains α and β, whereas CLIP's N and C terminus were acetylated and amidated, respectively. The corresponding mutations in the starting structures of DR1/C_W_ and DR1-βN82A/C_W_ were modelled atomically clash-free by substituting the βAsn82 (CLIP Met107) side-chain with the one of Ala (Trp) resulting in four different starting structures. The starting structures for 162 DR1-βT41A/C_W_ simulations were generated from the MSM of the DR1/C_W_ simulations by uniformly sampling in the kmeans-clustered (*k*=6≈ sqrt(no. of microstates in the largest connected MSM set)) space of the first three MSM eigenvectors. Each structure was aligned onto its principal axes and solvated with a 150 mM l^−1^ NaCl using VMD^58^. The resulting simulation boxes were about 70 × 80 × 120 Å contained 67,000 atoms, including 20,000 water molecules.

### MD simulations

Simulations were run using ACEMD^62^ with the ff99SB^63^ Amber force-field, an integration time-step of 4 fs and a hydrogen mass scaling factor of 4, a 1–4 scaling factor of 5/6, orthorhombic periodic boundary conditions and Particle Mesh Ewald electrostatics with 1 Å grid spacing, a 9 Å cutoff, switching at 7.5 Å, scale1–4 exclusion, full electrostatic frequency of 2 steps, fixed bonded interactions between heavy atoms and hydrogens (‘rigidbonds all'), and Langevin dynamics. In the beginning of the MD simulations, three replicates of each of the four starting structures were subjected to 5,000 steps of minimization, followed by 8ns isothermal-isobaric ensemble (NPT) equilibration with with 1 ps^−1^ Langevin damping, 1 atm Berendsen pressure and 800 fs relaxation time, and with harmonic, positional restraints on all protein heavy atoms (initial force constant: 5 kcal mol^−1^ Å^−2^). For the first 4ns in steps of 0.1 ns, these restraints were gradually reduced to zero, while the simulation temperature was gradually increased from 10 K to 310 K. For the remaining MD simulations, the pressure control was turned off (NVT- canonical ensemble), and the Langevin damping was set to 0.1 ps^−1^. Each of these remaining simulations, that is, of the six replicates for each DR1/C_W_ and DR1-βN82A/C_W_, we performed for about 1 μs. Each final restart point of these simulations was then replicated again (27 times), and simulated on the Titan supercomputer for another 550 ns each. Each of the 162 DR1-βN82A/C_W_ simulations was run on an in-house graphics-processing unit (GPU) cluster for 200–1,100 ns. The resulting MD simulation data set of 486 individual simulations with 275 μs total aggregated simulation time was then used for analysis.

Throughout all our MD simulations, we harmonically restrained the center of mass and the inertia tensor's off-diagonal elements of pMHCII's heavy atoms to zero. We implemented these restraints as an ACEMD plugin, which is available upon request. This strategy allowed us to perform our MD simulations with the periodic boundary conditions more efficiently, as the principle components of this intertia tensor had been aligned in the starting structures (see ‘MD set-up'). This gain in efficiency (∼50% speed-up for pMHCII) is due to the fact that an additional box size (and thus additional water molecules) is not necessary to accommodate for the rotational tumbling of pMHCII under periodic boundary conditions, as has been studied previously^64^,^65^. For pMHCII, the force constant values of these restraints (2 kcal mol^−1^ Å^−2^ and 2 kcal mol^−1^ Å^−4^, respectively) were carefully chosen, so that with a minimal bias, the minimal distances between pMHCII and each of its periodic images would not fluctuate on average over all three box dimensions by more than 0.8 Å during short MD test simulations (<50 ns). In the course of our MD simulations, these minimal distances were closely monitored not to be smaller than two times 9 Å, the electrostatic cutoff.

### Markov state modelling

MSMs^24^,^47^,^66^,^67^,^68^ were constructed with pyEMMA (www.pyemma.org)^26^ to arrive at a systematic analysis of the large amounts of simulation data. The data of DR1/C_W_ as well as the DR1-βN82A/C_W_, and DR1-αT41A/C_W_ mutants were transformed and discretized in the same way in order to arrive at models at which the effect of the mutant can be consistently compared: In particular, the MSMs for DR1/C_W_ and DR1-βN82A/C_W_ were computed from the combined discretized simulation set of DR1/C_W_ and DR1-βN82A/C_W_, whereas the MSM for DR1-αT41A/C_W_ was determined from the combined discretized simulation set of DR1/C_W_, DR1-βN82A/C_W_ and DR1-αT41A/C_W_. To this end, all pairwise Cα-coordinates of residues within 15 Å of βN82 in a simulated 3PDO conformation (that is, αH5-αA10, αM23-αD27, αE30-αF32, αF48-αE55 (not αR50), βR13-βL27 (not βN19), βR71-βR93 (not βR72, βn82)) were used as input coordinates to a time-lagged independent component analysis^69^,^70^. After the time-lagged independent component analysis transform, the three most important reaction coordinates were partitioned to 100 discrete states using k-means clustering. The simulations were assigned to this discrete state space and a reversible Markov model was estimated using the algorithm described in ref. 67. Based on implied timescales calculations^71^, a lag time of 200 ns was selected (Supplementary Fig. 10, left panels). The resulting Markov models were validated using a Chapman–Kolmogorov Test^67^,^72^ (Supplementary Fig. 10, right panels), and coarse-grained into three metastable states using PCCA++^73^ (Figs 1d and 3g) to represent metastable conformational transitions on the microsecond scale. Molecular graphics and movies were generated with the program PyMol^74^.

### Pairwise interaction analysis

Pairwise interactions and their significant differences between pairs of metastable states were detected with a modification of the system-wide Pairwise Interaction Analyser^75^,^76^: For every simulation frame assigned to a metastable conformation, a pairwise interaction between two sets of heavy atoms (for example, the heavy side-chain or backbone atoms a residue) is inferred to exist, if the two sets fulfill certain geometric criteria evaluated by the program HBPLUS^77^, or the program VMD^78^ (using a distance criterion provided in ref. 79). For a bootstrapped sample of 100 frames from an metastable state, the list of pairwise interactions were then combined into relative interaction frequencies, and the averages and standard deviations of these frequencies is computed for 50 of such bootstrapped samples. For each bootstrapped metastable state sample, entire trajectories are resampled with fixed k-means microstate definition and microstate-to-metastable state mappings and updated Markov model.

### Data availability

The crystal structures of DR1/C_W_ (4X5W) and DR1-βN82A/C_W_ (4X5X) have been deposited with the Protein Data Bank with accession codes 4X5W and 4X5X, respectively. The data that support the findings of this study are available from the corresponding authors upon request.

## Additional information

**How to cite this article:** Wieczorek, M. *et al*. MHC class II complexes sample intermediate states along the peptide exchange pathway. *Nat. Commun.* **7,** 13224 doi: 10.1038/ncomms13224 (2016).

## Supplementary Material

Supplementary Information Supplementary Figures 1 - 10 and Supplementary Tables 1 - 4

Supplementary Movie 1 Molecular movie illustrating the intrinsic, partial peptide dissociation pathway in the ground state MS3. The movie was extracted from a βN82A simulation. The molecular representation was chosen as in Figs. 1e, 3d, 4g. The representative simulations are filmed here at a frame rate of 10ns/1sec, and are smoothed with an averaging window of 11ns.

Supplementary Movie 2 Molecular movie illustrating a possible transition pathway from MS3 to the transition state candidate MS1, in which a destabilization of the β_1_-chain α-helix is followed by flipping out of the αW43 sidechain. The movie was extracted from a WT simulation. The molecular representation was chosen as in Figs. 1e, 3d, 4g. The representative simulations are filmed here at a frame rate of 10ns/1sec, and are smoothed with an averaging window of 11ns.

Supplementary Movie 3 Molecular movie illustrating a possible transition pathway from MS3 to the transition state candidate MS1, in which flipping out of the αW43 sidechain is followed by destabilization of the β_1_-chain α-helix. The movie was extracted from a βN82A simulation. The molecular representation was chosen as in Figs. 1e, 3d, 4g. The representative simulations are filmed here at a frame rate of 10ns/1sec, and are smoothed with an averaging window of 11ns.
