# Supplementary material for: MHC class II complexes sample intermediate states along the peptide exchange pathway
Source: Nat Commun. 2016 Nov 9;7:13224. doi: 10.1038/ncomms13224 (PMC5105163; doi:10.1038/ncomms13224)
Supplement: Supplementary Information — Supplementary Figures 1 - 10 and Supplementary Tables 1 - 4 [file ncomms13224-s1.pdf]

Supplementary information

Supplementary Figures

Supplementary Figure 1

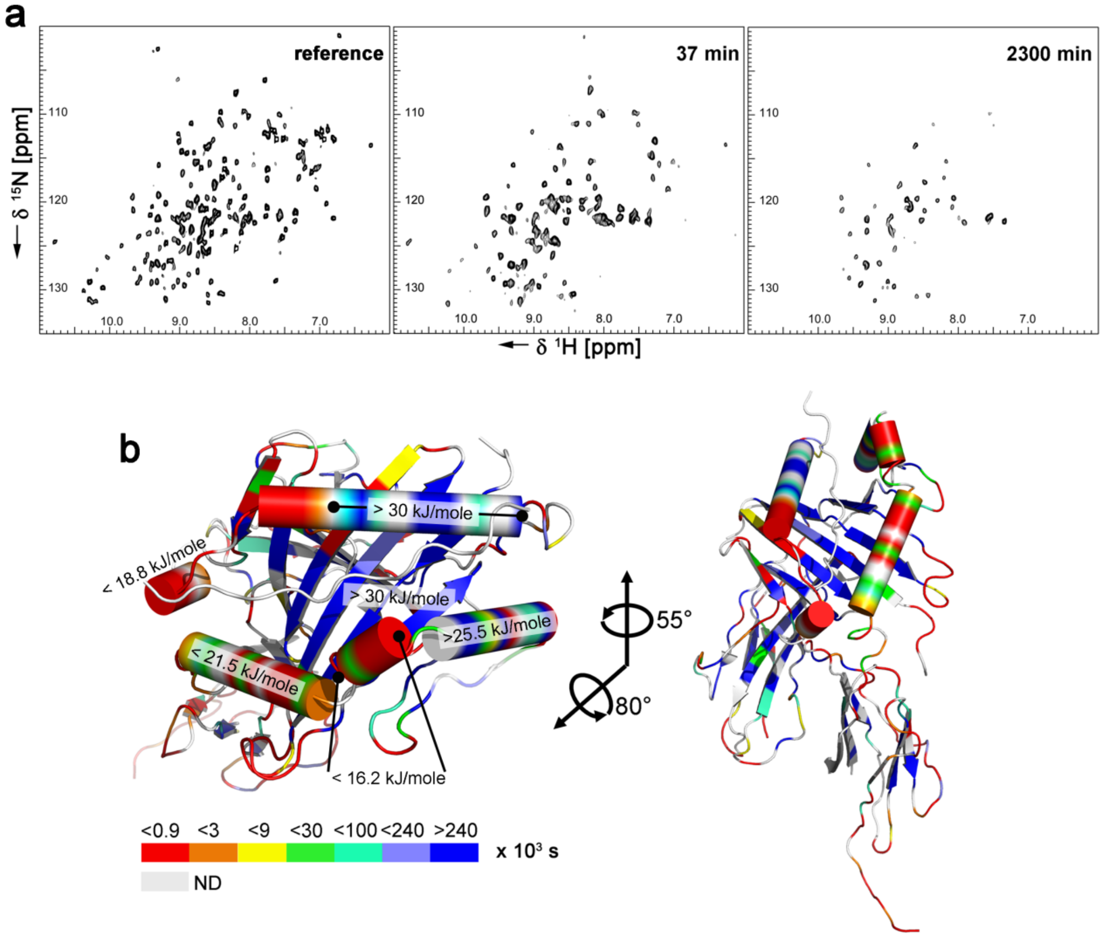

Supplementary Figure 1: NMR-derived H/D exchange of DR1/C$_W$. a) ^{1}H-^{15}N-HSQC spectra of ^{15}Nα-labeled DR1/C$_W$ in 10 % D_2O / 90 % H_2O and at different time points in 100 % D_2O. **b)** The backbone HDX pattern of DR1/C$_W$ reveals highly stable α1 and β1 β-sheets and a variable stability of the helical segments. No values where determined for residues colored white (ND). Average $\Delta G_{cl\text{-}op}$ values indicate increased flexibility of structural elements centered around the peptide´s N-terminus.

Supplementary Figure 2

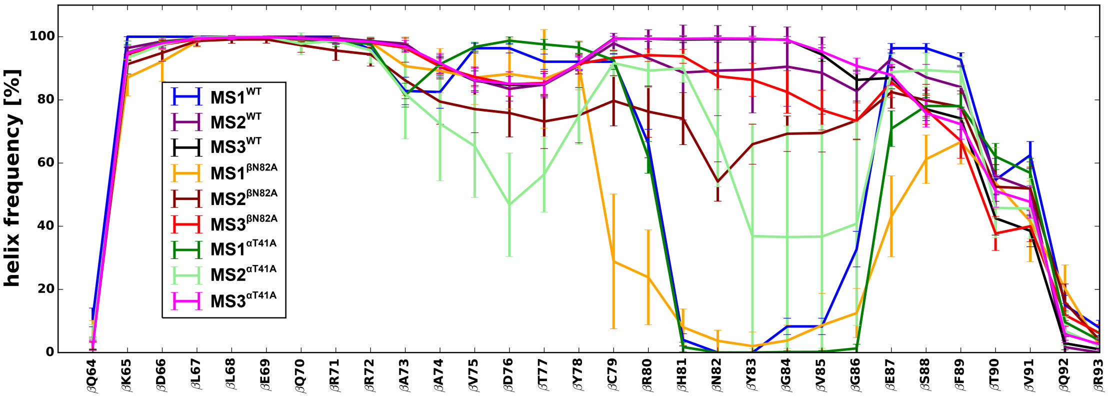

Supplementary Figure 2: Helix unfolding of βC79-βR93 is characteristic of metastable states $MS1^{WT}$, $MS1^{\beta N82A}$ and $MS1^{\alpha T41A}$ (cf. Figs. 1e, 3d, 4g, S7). Shown are average helix frequency plots for DR1 residues βQ64-βR93, as computed with the program VMD[57] from 50 bootstraps of each 100 samples. The error bars shown are standard deviations of the bootstrapped frequencies.

Supplementary Figure 3

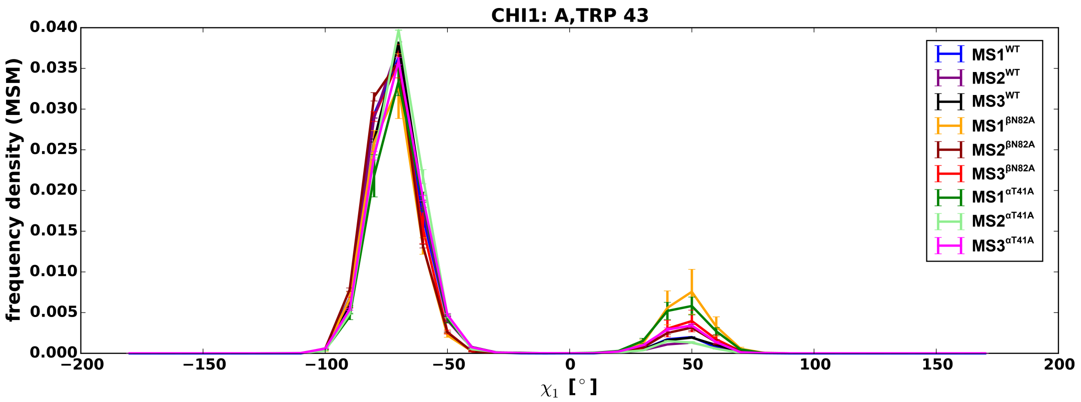

Supplementary Figure 3: αW43 χ_1 dihedral distributions plotted for each simulated metastable state presented in Figs. 1e, 3d, 4g, and S7. The error bars shown are standard deviations of the bootstrapped frequencies.

Supplementary Figure 4

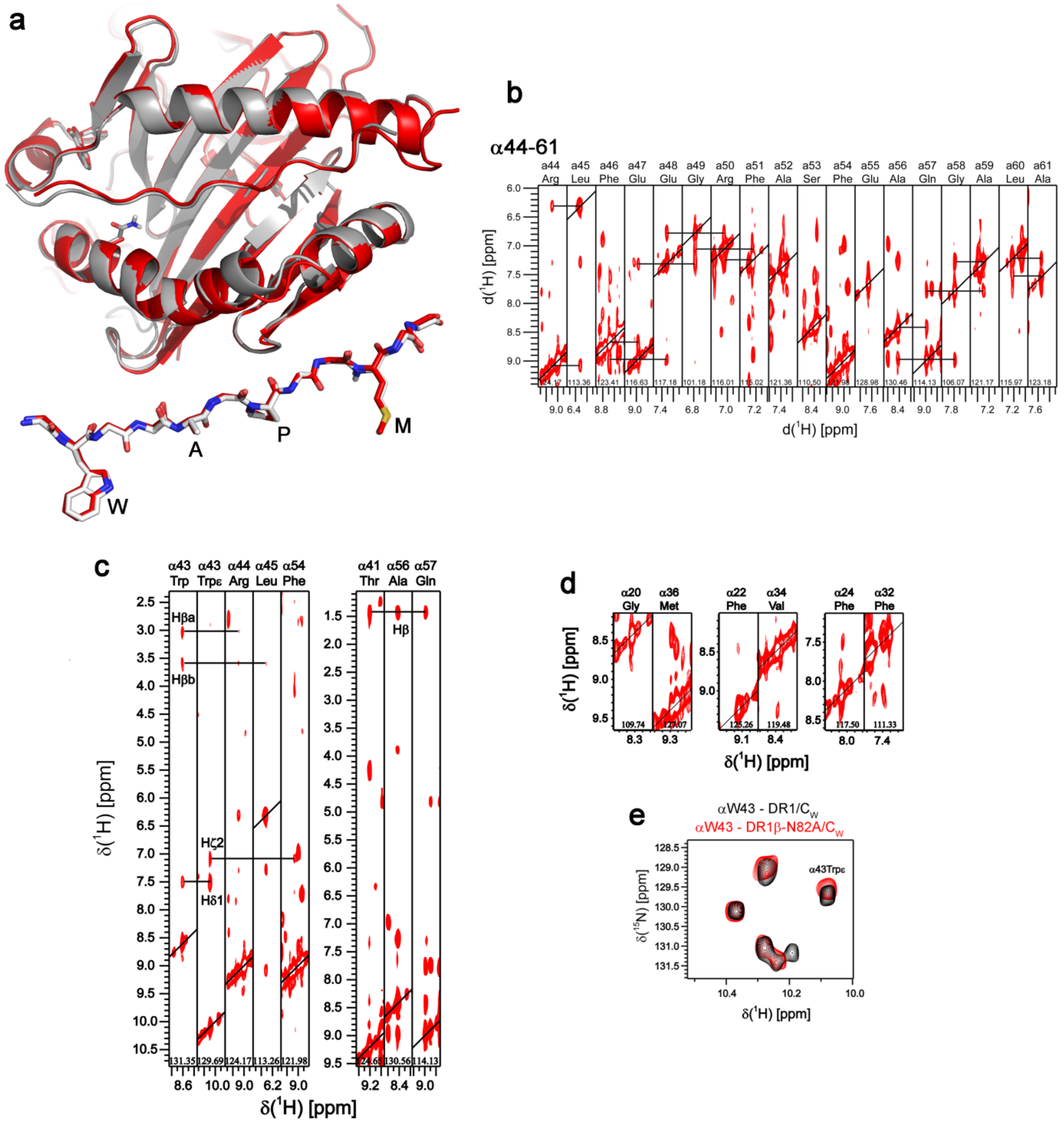

Supplementary Figure 4: Major conformation of DR1-βN82A/C_W reflects a canonical pMHCII fold. a) Top view of the structural alignment of DR1/C_W (grey) and DR1-βN82A/C_W (red). DRα W43 and β82 are represented as sticks. Shown underneath the structure is the bound peptide represented as sticks highlighting the sidechains occupying pocket P1 (Trp), P4 (Ala), P6 (Pro) and P9 (Met). **b, c, d)** NOESY-strips of ^{2}H/^{15}N-αβ-DR1-βN82A/C_W with selective labeling of ^{1}H-Ala in the α-chain and ^{1}H-Trp in α/β-chains reveal that **b)** The DM interface region maintains its fold as indicated by residues α44-61; **c)** There is no indication of a flipped out conformation of αTrp43; **d)** NOESY-strips of residues α20 and α36, α22 and α34, α24 and α32 show that β-sheets do arrange as in the DR1/C_W crystal structure. **e)** ^{1}H,^{15}N-TROSY-HSQC of the sidechain NH of αTrp43 in DR1/C_W and DR1-βN82A/C_W reveals no marked difference.

Supplementary Figure 5

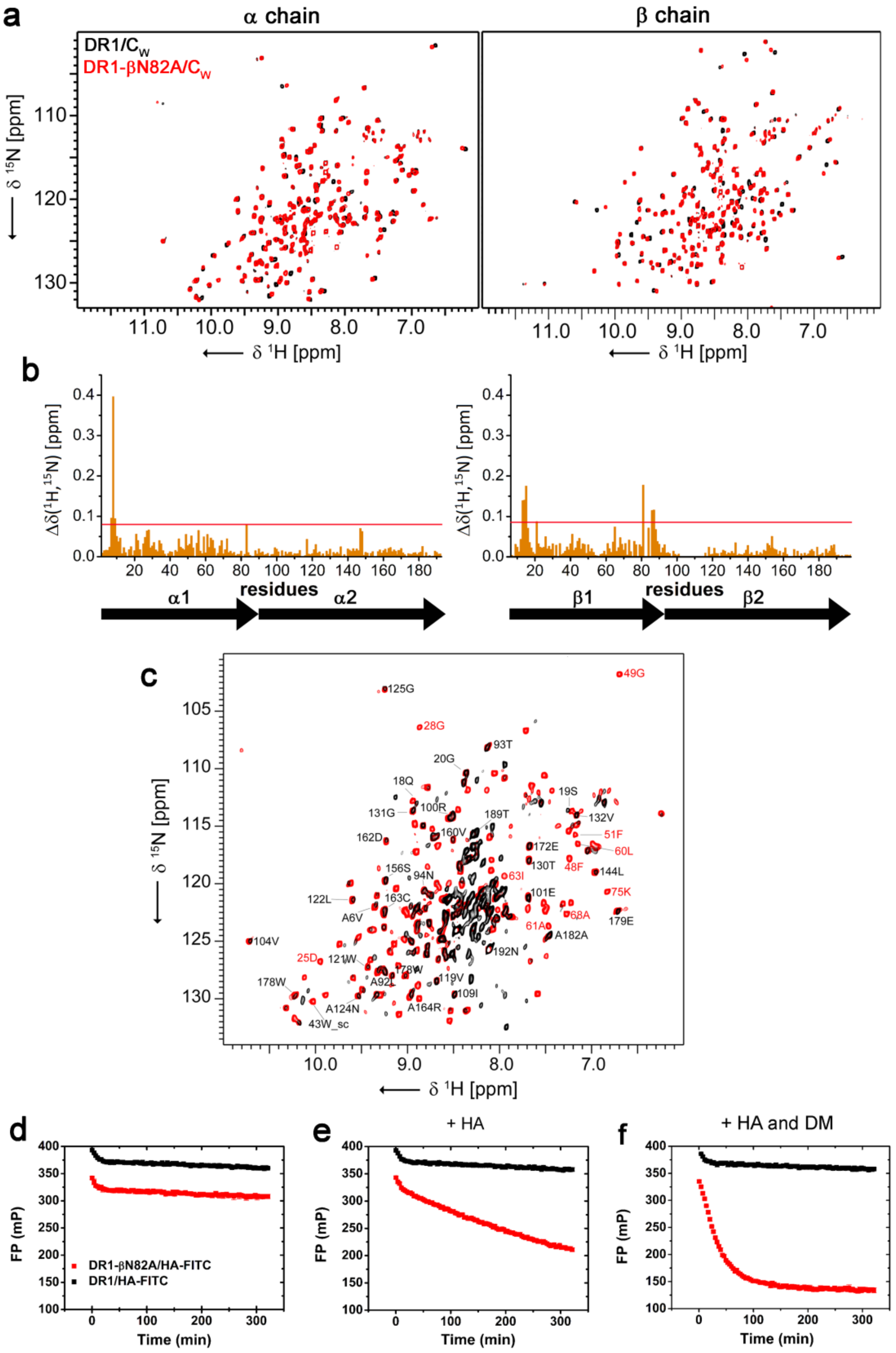

Supplementary Figure 5: In the presence of high-affinity peptide DR1-βN82A adopts both a loaded and a ligand-receptive conformation. a) Overlay of ^{1}H,^{15}N-TROSY-HSQC spectra of the α-chain (left) and β-chain (right) of DR1/C$_W$ and DR1-βN82A/C$_W$. **b)** Plot of chemical shift differences derived from (a). The red horizontal lines indicate the significance cutoff of 1SD above the average. **c)** Overlay of ^{1}H,^{15}N-TROSY-HSQC spectra of ^{15}Nα-labeled DR1-βN82A in the peptide-free form (black) and complexed with C$_W$ (red). **d)** Dissociation of preloaded HA-FITC from DR1 and DR1-βN82A, as measured by FP over time in the absence of unlabeled competitor and DM. **e)** Similar conditions as in (d), but in the presence of unlabeled HA. **f)** Similar conditions as in (e), but with the addition of DM. FP measurements were performed as triplicates and result from two independent experiments.

Supplementary Figure 6

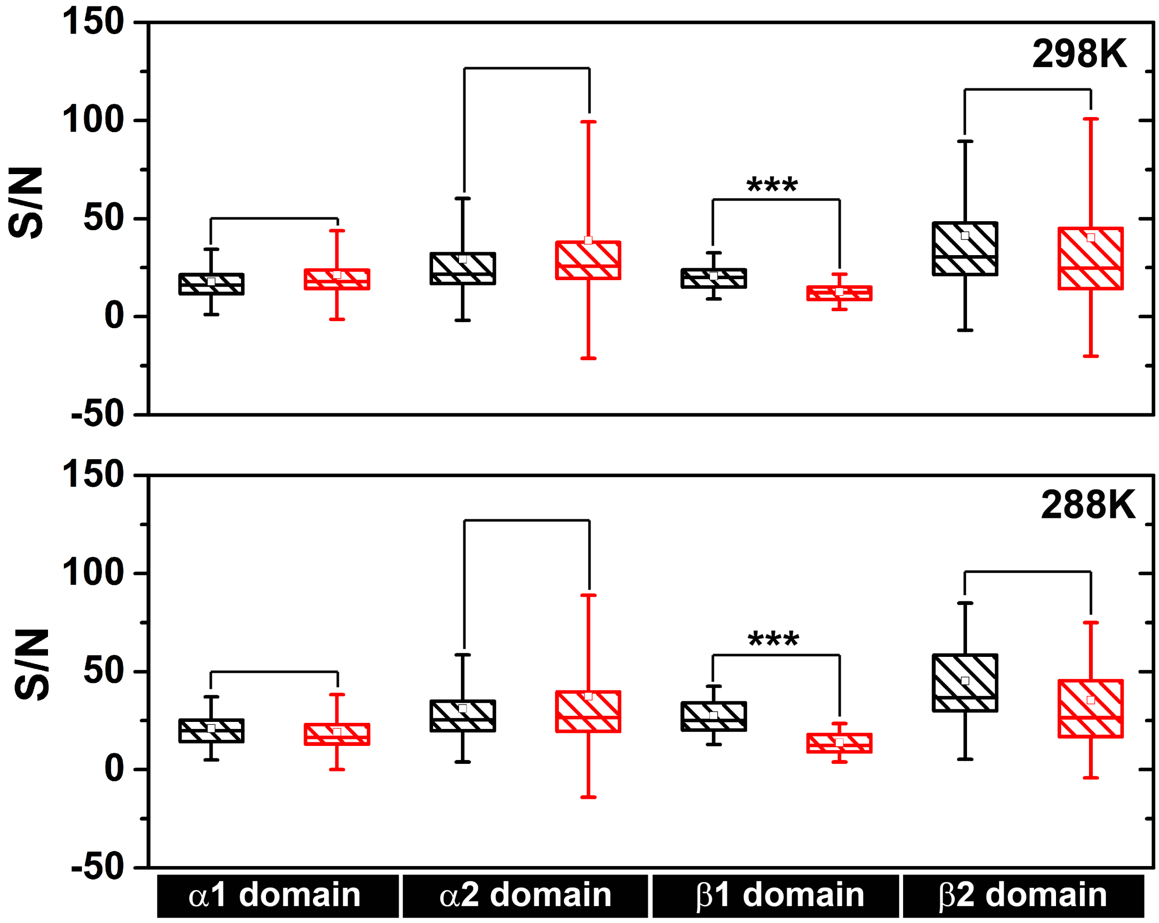

Supplementary Figure 6: Box plot of S/N ratios for backbone NH groups of the individual domains of DR1/C_W and DR1-βN82A/C_W measured at 25°C (298K, upper panel) and 15°C (288K, lower panel) showing the significant global increase in line broadening of the β1 domain in DR1-βN82A/C_W relative to the WT complex. In order to calculate S/N, peak-heights were determined from ^{1}H-^{15}N-TROSY-HSQC spectra of 50μM ^{15}N β-labeled DR1/C_w (144scans, noise level 2425 at 25°C and 4616 at 15°C), ^{15}N β-labeled 50μM DR1-βN82A/C_w (144 scans, noise level 2545 at 25°C and 4833 at 15°C), 60μM ^{15}N α-labeled DR1/C_w (144 scans, noise level of 4955 at 25°C and 4711 at 15°C) and 35μM ^{15}N α-labeled DR1/C_w (340 scans to account for the difference in protein concentration, noise level of 1978 for 25°C and 3679 for 15°C). *** means $p < 0.001$. S/N analyses were performed for residues composing the folded domains (α1 residues 2-85, α2 residues 86-189, β1 residues 6-95 and β2 residues 96-193).

Supplementary Figure 7

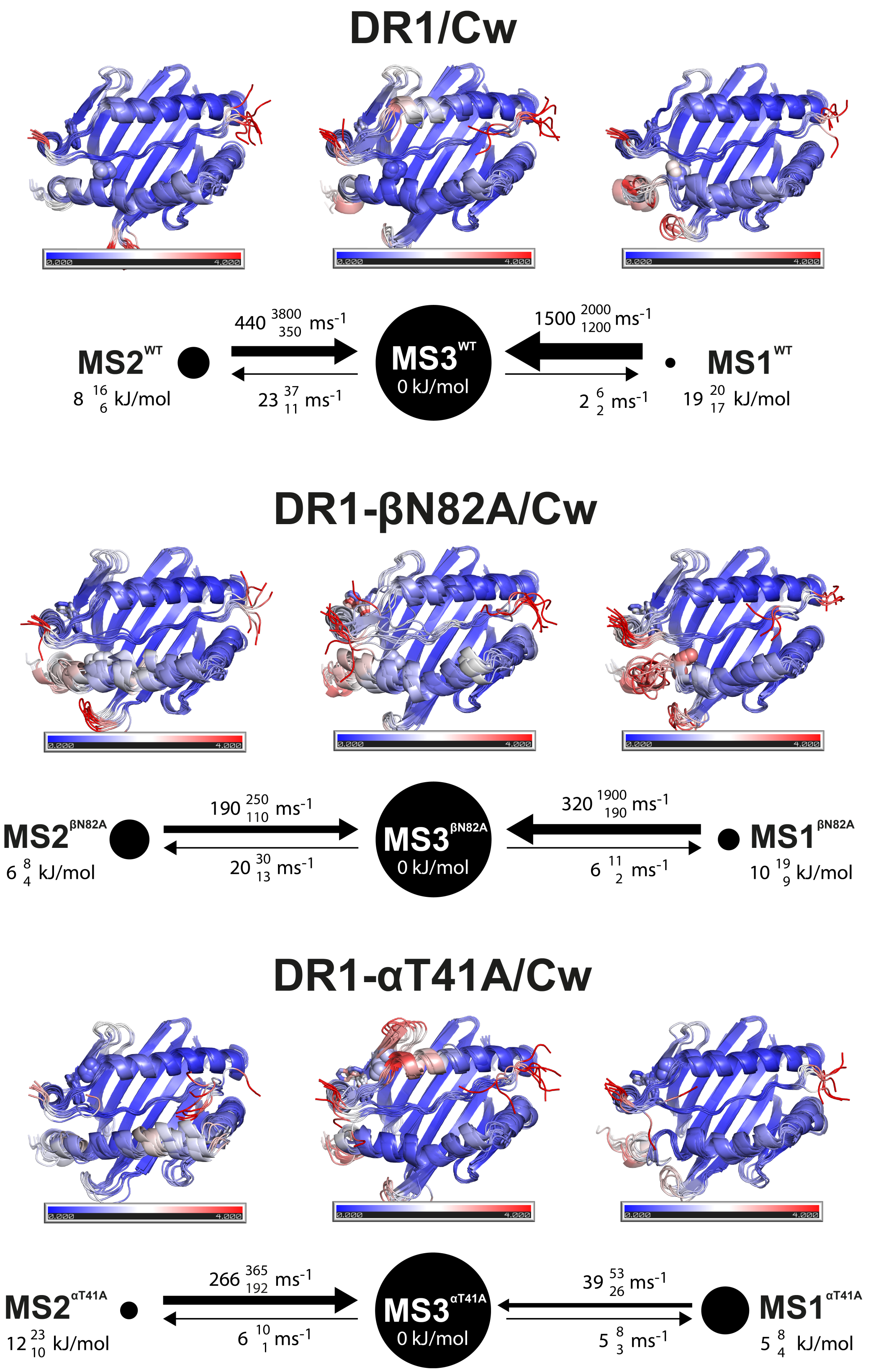

Supplementary Figure 7: The metastable states of MHCII adopt different, intrinsic conformational dynamics. Kinetic maps are shown as in Figs. 1d/3g/4g, but here residues along the MHCII ribbon conformations of each MS are colored based on their iterative fit root-mean-square-fluctuations (RMSF), i.e. from 0Å (blue) over 2Å (white) to ≥4Å (red).

Supplementary Figure 8

a

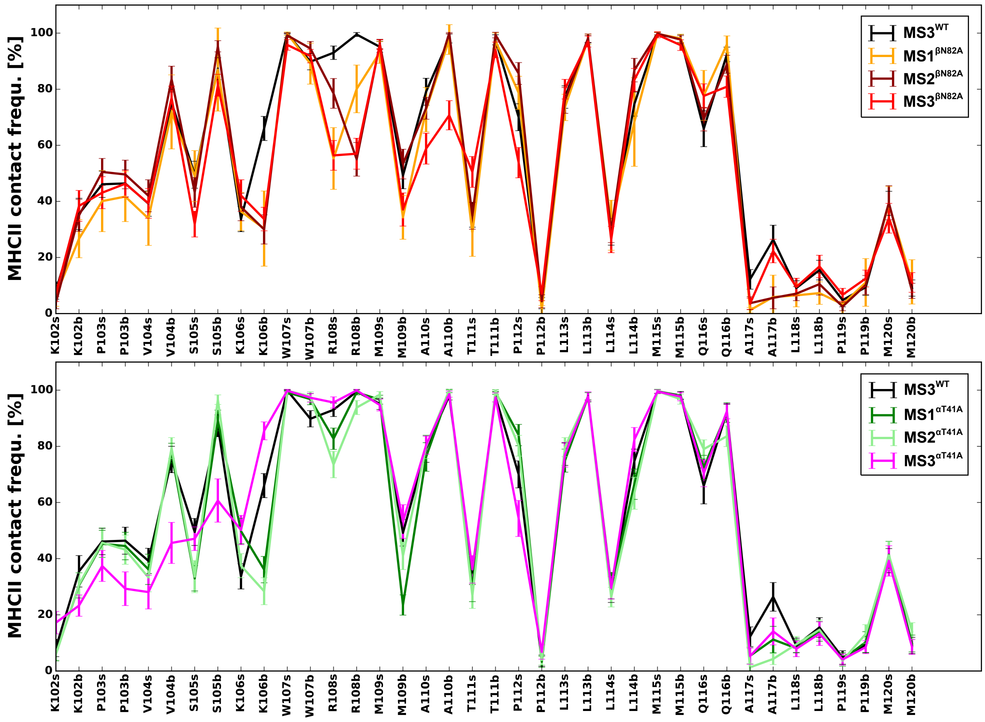

b

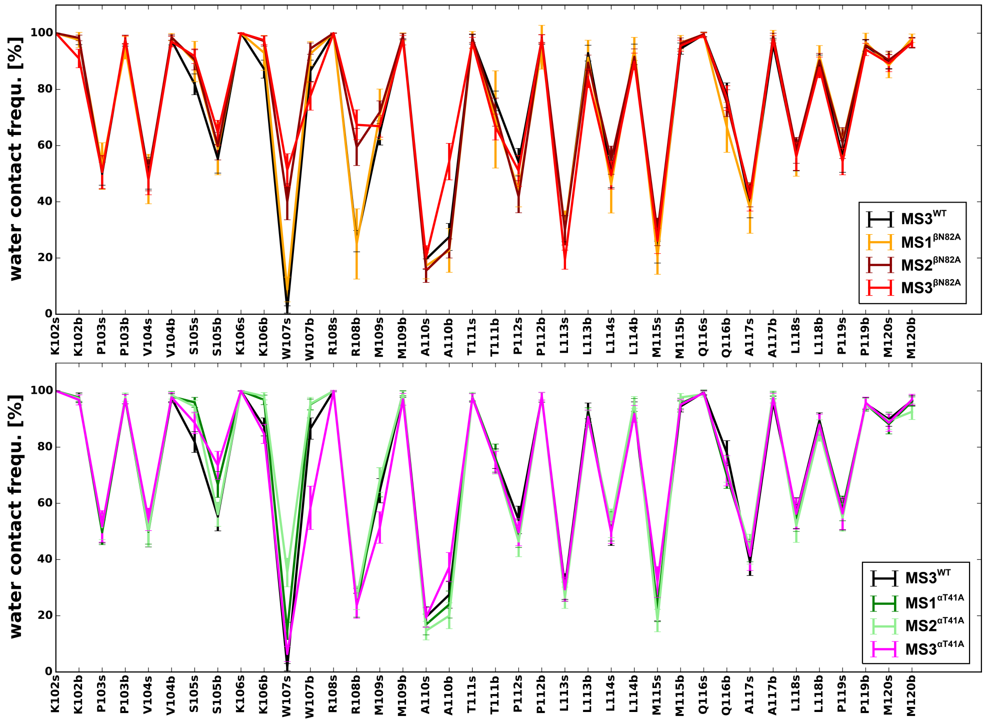

Supplementary Figure 8: The core of the CLIP ligand (residues P1 to P9) of the simulated DR1-βN82A/C_W is more likely to partially dissociate and allow water penetration than in DR1/C_W while in DR1-αT41A/C_W it remains stable. Shown are average contact frequencies of specific CLIP residues ("s" for side chain, "b" for backbone atoms) with (a) any MHCII residue (b) and any water molecule. For each plot, error bars (bootstrapped standard errors) were computed for the average frequency of 50 bootstraps with 100 samples each.

Supplementary Figure 9

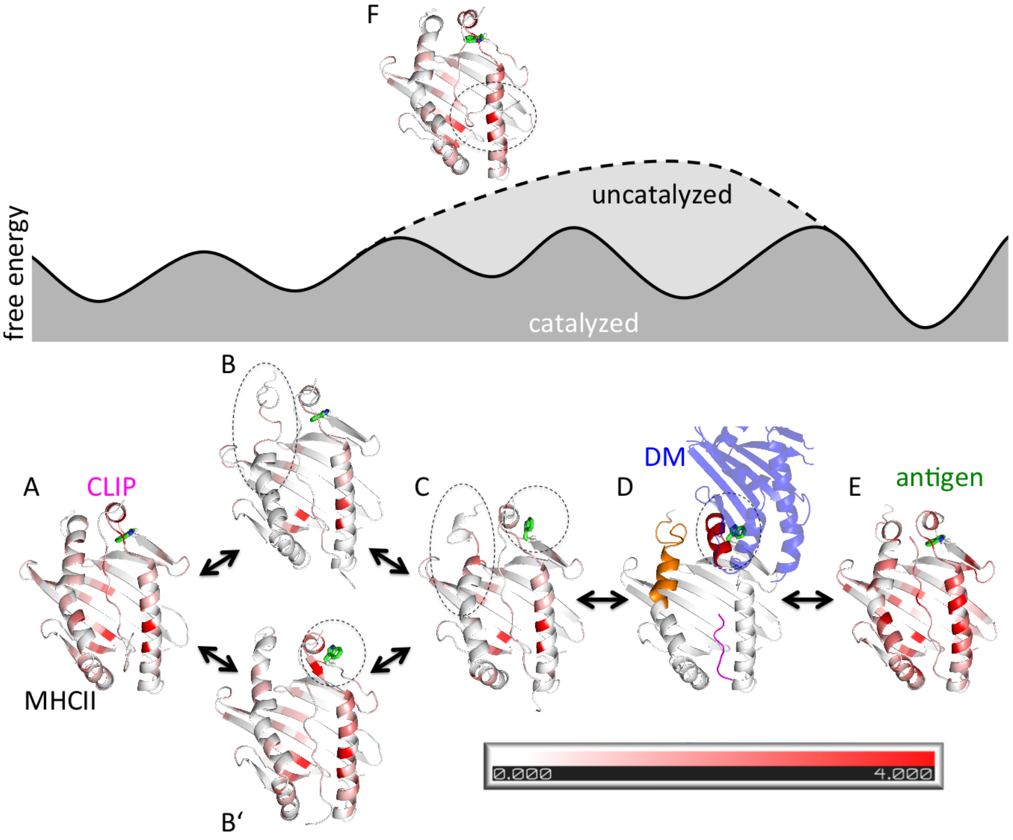

Supplementary Figure 9: Structural differences compared to the DM-bound crystal structure. States of the uncatalyzed and catalyzed peptide-exchange pathways (as shown in Fig. 5) are compared to the DM-bound crystal structure of DR1 (panel D). In all other structures (A-D, E-F), residues are colored according to their differences in polar interactions with other residues (white: 0 differences to red: ≥4 differences). For panels A-C,F, we define a significant difference in a particular interaction *i* (c.f. Methods), if $| f_{i,MS} - f_{i,4FQX} | - 16\sqrt{(\Delta f_{i,MS}^2 + \Delta f_{i,4FQX}^2)} > 0$, where $f_{i,MS}$ is the frequency of interaction *i* in a particular metastable state MS, $f_{i,4FQX}=1$ ($f_{i,4FQX}=0$), if the interaction *i* is present (not present) in 4FQX, and $\Delta f_{i,MS}$ is the interaction frequency's standard error in MS ($\Delta f_{i,4FQX} \equiv 0$). For panel E, we consider any difference in polar interaction between the 1DLH and 4FQX crystal structures.

Supplementary Figure 10

a

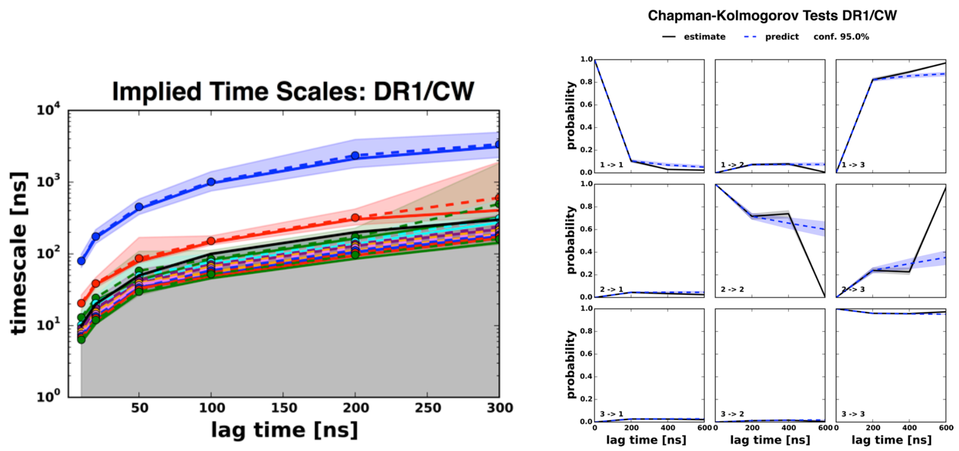

b

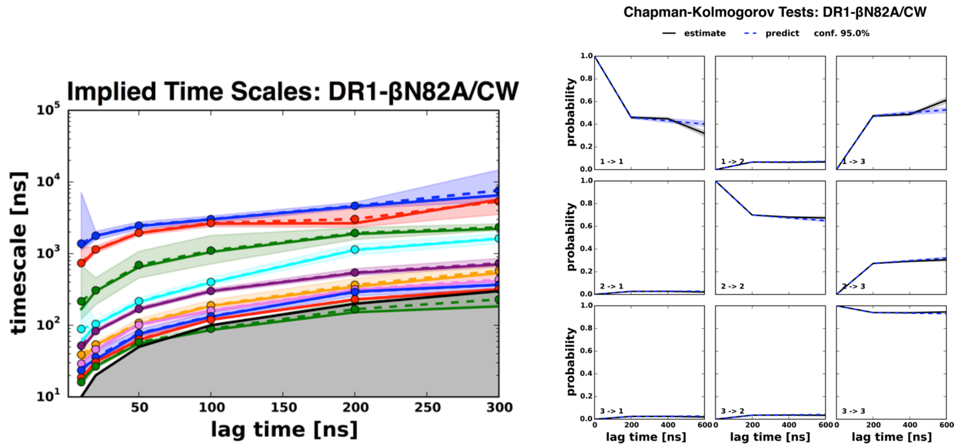

c

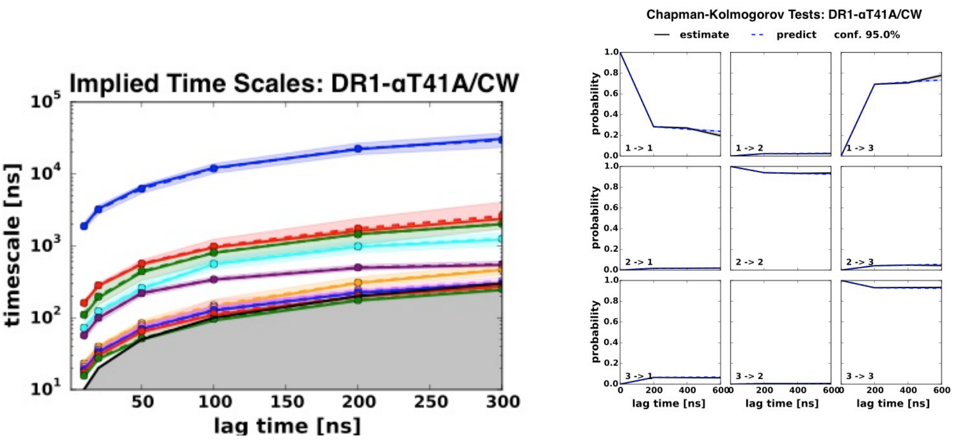

Supplementary Figure 10: **Markov State Model validations**. (left panels) Implied time scales (ITS) for DR1/C$_W$ (a), DR1-βN82A/C$_W$ (b), and DR1-αT41A/C$_W$ (c) are plotted against lag time. The 1st, 2nd, 3rd, … slowest ITS are colored blue, red, green, … Solid lines mark ITS of the maximum likelihood Markov Model, whereas dashed lines with shaded areas represent mean and 95% credibility interval computed by Bayesian sampling. A lag time of 200 ns was selected. (right panels) Chapman-Kolmogorov tests, comparing the transition probabilities between metastable states predicted by the MSM at longer lag times (blue) with the ones predicted by independent MSM estimation at that lag time (black). Note that only few trajectories are available at 600 ns, such that the statistical error of the MSM estimate at 600 ns could not be computed in panel a, right.

Supplementary Tables

Complex	Thermal stability
**DR1/C**	**80.0 ± 0.9**
**DR1/C_W**	**84.6 ± 0.6*** **
**DR1-αN82A/C_W**	**65.8 ± 2.3*** **

Supplementary Table 1: Denaturation temperature of complexes investigated in Figure 1-3. Experiments were performed as triplicates and result from n=5 independent experiments for DR1/C, n=5 for DR1/C_W and n=4 independent experiments for DR1-βN82A/C_W. Significance test (t-Test) was performed for DR1/C vs. DR1/C_W and for DR1/C_W vs. DR1-βN82A/C_W. Shown are the mean values ± s.d. ***: p< 0.001.

	**DR1/C_W**	**DR1-βN82A- β-$(G_4S)_3$-C_W**
**Data collection**		
**Wavelength (Å)**	0.91841	0.91841
**Resolution range (Å)**	34.7 - 1.34 (1.39 - 1.34)	34.25 - 3.2 (3.31 - 3.2)
**Space group**	$C222_1$	$P2_1$
**Unit cell**		
**a, b, c (Å)**	73.78 90.85 138.79	57.82 86.47 94.11
**α, β, γ (°)**	90 90 90	90 103.96 90
**Unique reflections**	102530 (8894)	14831 (1449)
**Multiplicity**	4.0 (3.1)	3.1 (3.1)
**Completeness (%)**	98.16 (85.77)	98.94 (98.91)
I/σI	15.41 (2.09)	5.68 (2.26)
**R_{merge} (%)**	4.9 (49.2)	18.6 (52.3)
**Data refinement**		
**R_{work}/R_{free} (%)**	14.6/17	22/26.2
**Number of atoms**	3928	6130
protein	3404	6130
**water**	524	0
**RMS(bonds)**	0.016	0.002
**RMS(angles)**	1.56	0.69
**Ramachandran** statistics **(%)**		
favored	98	98
**allowed**	2	2
**outliers**	0	0
**Average B-factor**	20.70	44.10
protein	19.00	44.10
water	31.90	

Supplementary Table 2: Data collection and refinement statistics. Statistics for the highest-resolution shell are shown in parentheses.

Alpha chain				Beta chain			
Residue	$t_{½}$ [s]*10^3	Protection factor[a]	ΔG [kJ*mole^{-1}]	Residue	$t_{½}$ [s]*10^3	Protection factor[a]	ΔG [kJ*mole^{-1}]
αV6	9.1 ± 1.3	4.8 ± 0.7	21.9 ± 0.4	βF18	4.7 ± 1.5	8.9 ± 2.9	23.4 ± 0.8
αE11	186.3 ± 45.1	63.0 ± 15.23	28.5 ± 0.6	βR39	161.2 ± 17.7	133.1 ± 14.6	30.4 ± 0.3
αF12	214.1 ± 40.2	108.3 ± 22.3	29.9 ± 0.5	βD41	91.4 ± 28.0	92.5 ± 28.4	29.5 ± 0.8
αQ18	3.1 ± 0.7	6.2 ± 1.6	22.5 ± 0.7	βS42	64.4 ± 14.9	78.3 ± 18.1	29.1 ± 0.6
αS19	5.2 ± 0.8	17.3 ± 2.7	25.2 ± 0.4	βG45	29.2 ± 8.9	24.2 ± 7.4	26:0 ± 0.8
αD27	4.0 ± 1.1	3.5 ± 1.1	21.0 ± 0.8	βT51	15.4 ± 2.9	7.9 ± 1.5	23.1 ± 0.5
αE30	6.7 ± 1.3	1.6 ± 0.4	19.0 ± 0.6	βG54	31.1 ± 8.7	49.2 ± 11.5	27.9 ± 0.6
αV34	127.0 ± 21.1	86.6 ± 17.8	29.3 ± 0.5	βR55	55.6 ± 9.7	44.8 ± 7.8	27.6 ± 0.4
αD35	86.1 ± 12.9	38.2 ± 5.8	27.2 ± 0.4	βE59	7.4 ± 1.8	2.4 ± 0.6	20.1 ± 0.6
αE40	14.5 ± 2.0	6.9 ± 1.0	22.8 ± 0.4	βY60	10.6 ± 2.0	4.0 ± 0.8	21.4 ± 0.5
αR44	68.3 ± 19.4	35.0 ± 11.8	27.0 ± 0.9	βQ64	29.1 ± 6.6	49.2 ± 11.2	27.9 ± 0.6
αF48	2.8 ± 0.8	3.3 ± 0.9	20.9 ± 0.7	βL68	13.9 ± 2.3	2.3 ± 0.4	19.9 ± 0.4
αA52	0.9 ± 0.6	1.4 ± 1.0	18.6 ± 1.9	βR71	10.6 ± 2.8	18.6 ± 5.0	25.3 ± 0.7
αL60	3.5 ± 1.0	1.4 ± 0.4	18.7 ± 0.7	βA73	2.0 ± 0.6	2.5 ± 0.7	20.1 ± 0.8
αN62	40.2 ± 4.0	63.2 ± 6.6	28.5 ± 0.3	βA74	17.5 ± 3.5	9.5 ± 1.9	23.6 ± 0.5
αA64	57.0 ± 12.9	20.4 ± 4.6	25.6 ± 0.6	βR80	19.1 ± 3.7	93.7 ± 18.3	29.5 ± 0.5
αE71	58.4 ± 8.8	9.0 ± 1.5	23.5 ± 0.4	βG84	9.2 ± 1.0	9.9 ± 1.0	23.7 ± 0.3
αR76	108.8 ± 16.2	318.0 ± 50.4	32.7 ± 0.4	βV85	47.5 ± 5.6	35.3 ± 4.2	27.0 ± 0.3
αS77	6.0 ± 1.7	27.2 ± 7.6	26.3 ± 0.7	βG86	7.6 ± 1.4	6.3 ± 1.2	22.5 ± 0.5
αT80	1.1 ± 0.5	0.4 ± 0.2	15.8 ± 1.1	βE87	2.7 ± 0.4	2.7 ± 0.4	20.4 ± 0.4
αT93	2.2 ± 0.5	5.3 ± 1.2	22.1 ± 0.6	βF89	11.0 ± 2.1	10.6 ± 2.0	23.9 ± 0.5
αN94	55.9 ± 16.0	236.6 ± 73.5	31.9 ± 0.8	βT90	21.8 ± 5.8	12.7 ± 3.4	24.4 ± 0.7
αS95	4.2 ± 0.5	9.3 ± 1.1	23.6 ± 0.3	βQ92	2.9 ± 0.4	3.6 ± 0.5	21.1 ± 0.4
αE101	3.5 ± 0.4	0.8 ± 0.1	17.3 ± 0.3	βR93	27.5 ± 6.8	48.4 ± 11.9	27.8 ± 0.6
αN103	73.3 ± 9.4	72.9 ± 9.3	28.9 ± 0.3	βG121	50.8 ± 12.3	104.7 ± 25.3	29.8 ± 0.6
αV104	67.2 ± 13.3	19.5 ± 4.2	25.5 ± 0.5	βI127	90.8 ± 10.4	23.2 ± 2.7	25.9 ± 0.3
αK126	92.0 ± 11.3	56.0 ± 8.0	28.2 ± 0.4	βE128	98.4 ± 16.5	15.2 ± 2.5	24.8 ± 0.4
αV128	25.9 ± 4.9	8.6 ± 1.7	23.4 ± 0.5	βW131	247.6 ± 54.0	122.5 ± 26.7	30.2 ± 0.6
αT129	2.2 ± 0.4	2.0 ± 0.4	19.6 ± 0.5	βN134	1.7 ± 0.3	6.8 ± 1.2	22.8 ±0.5
αR140	1.7 ± 1.0	8.6 ± 4.3	23.4 ± 1.3	βK139	134.5 ± 36.1	120.7 ± 32.4	30.2 ± 0.7
αF148	127.9 ± 17.9	340.0 ± 47.2	32.8 ± 0.4	βQ149	2.3 ± 0.4	5.7 ± 1.1	22.3 ± 0.5
αY150	23.7 ± 8.0	34.8 ± 11.3	27.0 ± 0.8	βM160	78.8 ± 18.4	37.3 ± 8.7	27.1 ± 0.6
αV160	4.2 ± 0.5	1.2 ± 0.3	18.2 ± 0.7	βE169	7.9 ± 1.6	2.3 ± 0.5	19.0 ± 0.5
αE166	231.0 ± 43.4	346.7 ± 78.9	32.9 ± 0.6	βV170	2.8 ± 0.4	0.7 ± 0.1	16.8 ± 0.4
αL174	11.4 ± 2.0	2.0 ± 0.4	19.6 ± 0.5	βV180	1.4 ± 0.4	0.6 ± 0.2	16.6 ± 0.7
αK176	90.2 ± 12.3	253.2 ± 38.7	32.1 ± 0.4	βL184	1.2 ± 0.1	0.4 ± 0.0	15.2 ± 0.2
α178	34.3 ± 8.7	51.2 ± 7.0	28.0 ± 0.4	βV186	52.4 ± 11.0	13.1 ± 2.8	24.4 ± 0.5
				βW188	49.4 ± 11.2	27.2 ± 6.2	26.3 ± 0.6

[a:] The intrinsic dissociation rates required to calculate the protection factors were derived from the Sphere server (http://www.fccc.edu/research/labs/roder/sphere/sphere.html). $k_{obs} = t_{1/2}^{-1}$. Errors result from curve fitting.

Supplementary Table 3: NMR-derived kinetic and thermodynamic constants for backbone H/D exchange of α and β residues in DR1/C_W.

	DR1/C_W			DR1-βN82A/C_W		
**Residue**	***t*** **½ [s]*10[3]**	**Protection factor[a]**	**ΔG [kJ*mole^{-1}]**	***t*** **½ [s]*10[3]**	**Protection factor[a]**	**ΔG [kJ*mole^{-1}]**
αQ18	3.1 ± 0.8	6.7 ± 1.6	22.7 ± 0.6	47.3 ± 9.0	96.4 ± 18.3	29.6 ± 0.5
αS19	5.2 ± 0.8	17.3 ± 2.7	25.2 ± 0.4	19.3 ± 3.8	64.7 ± 12.7	28.6 ± 0.5
αF22	<0.9	<0.6	<16.6	9.2 ± 2.6	6.4 ± 1.8	22.6 ± 0.7
αD27	4.0 ± 1.1	3.5 ± 1.1	21 ± 0.8	<0.9	<0.8	<17.2
αE30	6.7 ± 1.3	1.6 ± 0.4	19 ± 0.6	<0.9	<0.15	<12.9
αI63	>240	>106.3	>29.8	92.2 ± 31.9	40.8 ± 14.1	27.4 ± 0.9
αS77	6.0 ± 1.7	27.2 ± 7.6	26.3 ± 0.7	19.7 ± 2.8	89.5 ± 12.8	29.4 ± 0.4
αT80	1.1 ± 0.5	0.4 ± 0.2	15.8 ± 1.1	8.2 ± 2.5	3.5 ± 1.1	21.1 ± 0.8
αT93	2.2 ± 0.5	5.3 ± 1.2	22.1 ± 0.6	5.7 ± 1.4	14.0 ± 3.5	24.6 ± 0.7
αE101	3.5 ± 0.4	0.8 ± 0.1	17.3 ± 0.3	11.1 ± 1.9	2.7 ± 0.5	20.4 ± 0.4
αV128	25.9 ± 4.9	8.6 ± 1.7	23.4 ± 0.5	76.5 ± 10.7	25.4 ± 3.6	26.2 ± 0.4
βF18	4.7 ± 1.5	8.9 ± 2.9	23.4 ± 0.8	<0.9	<1.7	<19.2
βE46	>240	>94.9	>29.6	80.0 ± 26.3	31.6 ± 10.4	26.7 ± 0.8
βG54	37.1 ± 8.7	49.2 ± 11.5	27.9 ± 0.6	14.6 ± 3.0	19.3 ± 5.2	25.4 ± 0.7
βR55	55.6 ± 9.7	44.8 ± 7.8	27.6 ± 0.4	16.4 ± 4.7	13.2 ± 3.8	24.5 ± 0.7
βE59	7.4 ± 1.8	2.4 ± 0.6	20.1 ± 0.6	<0.9	<0.3	<14.6
βY60	10.6 ± 2.0	4.0 ± 0.8	21.4 ± 0.5	3.2 ± 0.8	1.2 ± 0.3	18.3 ± 0.6
βA74	17.5 ± 3.5	9.5 ± 1.9	23.6 ± 0.5	9.5 ± 1.8	5.2 ± 1.0	22.1 ± 0.5
βR80	19.1 ± 3.7	93.7 ± 18.3	29.5 ± 0.5	<0.9	<4.4	<21.6
βG84	9.2 ± 1.0	9.9 ± 1.0	23.7 ± 0.3	<0.9	<1.0	<17.7
βV85	47.5 ± 5.6	35.3 ± 4.2	27:0 ± 0.3	<0.9	<0.7	<16.8
βG86	7.6 ± 1.4	6.3 ± 1.2	22.5 ± 0.5	<0.9	<0.7	<17.1
βE87	2.7 ± 0.4	2.7 ± 0.4	20.4 ± 0.4	<0.9	<0.9	<17.5
βF89	11.0 ± 2.1	10.6 ± 2.0	23.9 ± 0.5	<0.9	<0.9	<17.4
βT90	21.8 ± 5.8	12.7 ± 3.4	24.4 ± 0.7	<0.9	<0.5	<16.2
βQ92	2.9 ± 0.4	3.6 ± 0.5	21.1 ± 0.4	<0.9	<1.1	<18.1
βR93	2.2 ± 0.5	5.3 ± 1.2	22.1 ± 0.6	<0.9	<1.6	<19.0
βI127	90.8 ± 10.4	23.2 ± 2.7	25.9 ± 0.3	1.8 ± 0.4	0.5 ± 0.1	15.8 ± 0.5
βE128	98.4 ± 16.5	15.2 ± 2.5	24.8 ± 0.4	21.4 ± 3.4	3.3 ± 0.5	20.9 ± 0.4
βN150	>240	>952	>35.5	28.1 ± 7.7	111.3 ± 30.7	30.0 ± 0.7
βM160	78.8 ± 18.4	37.3 ± 8.7	27.1 ± 0.6	9.6 ± 1.6	4.5 ± 0.7	21.7 ± 0.4
βV175	>240	>60	>28.4	96.5 ± 8.6	24.1 ± 2.1	26.0 ± 0.2
βE176	>240	>380.8	>33.1	113.4 ± 18.8	179.9 ± 29.9	31.2 ± 0.4

[a]: The intrinsic dissociation rates required to calculate the protection factors were derived from the sphere server (http://www.fccc.edu/research/labs/roder/sphere/sphere.html). $k_{obs} = t_{1/2}^{-1}$. Errors result from curve fitting. Table for NMR-derived kinetic and thermodynamic constants for backbone H/D exchange of α and β residues in DR1-βN82A/C_W is shown in the Appendix (Supplementary Table 5).

Supplementary Table 4: Comparison of kinetic and thermodynamic rates and constants for backbone H/D exchange between DR1/C_W and DR1-βN82A/C_W.
